# Effect of the Cross-Linking Density on the Swelling and Rheological Behavior of Ester-Bridged β-Cyclodextrin Nanosponges

**DOI:** 10.3390/ma14030478

**Published:** 2021-01-20

**Authors:** Gjylije Hoti, Fabrizio Caldera, Claudio Cecone, Alberto Rubin Pedrazzo, Anastasia Anceschi, Silvia Lucia Appleton, Yousef Khazaei Monfared, Francesco Trotta

**Affiliations:** 1 Department of Chemistry, University of Torino, Via P. Giuria 7, 10125 Torino, Italy; fabrizio.caldera@unito.it (F.C.); claudio.cecone@unito.it (C.C.); alberto.rubinpedrazzo@unito.it (A.R.P.); anastasia.anceschi@stiima.cnr.it (A.A.); silvialucia.appleton@unito.it (S.L.A.); yousef.khazaeimonfared@unito.it (Y.K.M.); francesco.trotta@unito.it (F.T.); 2 CNR-STIIMA, Istituto di Sistemi e Tecnologie Industriali Intelligenti per il Manifatturiero Avanzato, Consiglio Nazionale delle Ricerche, C.so Pella 16, 13900 Biella, Italy

**Keywords:** β-cyclodextrin nanosponges, swelling capacity, cross-linking density, Flory–Rehner theory, rheology

## Abstract

The cross-linking density influences the physicochemical properties of cyclodextrin-based nanosponges (CD-NSs). Although the effect of the cross-linker type and content on the NSs performance has been investigated, a detailed study of the cross-linking density has never been performed. In this contribution, nine ester-bridged NSs based on β-cyclodextrin (β-CD) and different quantities of pyromellitic dianhydride (PMDA), used as a cross-linking agent in stoichiometric proportions of 2, 3, 4, 5, 6, 7, 8, 9, and 10 moles of PMDA for each mole of CD, were synthesized and characterized in terms of swelling and rheological properties. The results, from the swelling experiments, exploiting Flory–Rehner theory, and rheology, strongly showed a cross-linker content-dependent behavior. The study of cross-linking density allowed to shed light on the efficiency of the synthesis reaction methods. Overall, our study demonstrates that by varying the amount of cross-linking agent, the cross-linked structure of the NSs matrix can be controlled effectively. As PMDA βCD-NSs have emerged over the years as a highly versatile class of materials with potential applications in various fields, this study represents the first step towards a full understanding of the correlation between their structure and properties, which is a key requirement to effectively tune their synthesis reaction in view of any specific future application or industrial scale-up.

## 1. Introduction

Hydrogels are chemically or physically three-dimensional nanoporous polymeric networks [1] (Figure S1, Supplementary Material). They contain cross-links that avoid the dissolution of the hydrophilic polymer chains into the aqueous phase [2]. As a result, they can swell in aqueous media rapidly [1] and undergo hydrolysis easily [3]. Hydrogels can be referred to as physical when polymer chains are connected by electrostatic forces, hydrogen bonds, hydrophobic interactions, or chain entanglements or as chemical when they are connected by covalent bonds [4]. The unique structure of cyclodextrins (CDs) has a great potential in the preparation of hydrogels [5] for pharmaceutical applications, in which they act as drug [6,7,8,9,10] and protein delivery systems [11]. Therefore, for many years, CDs have fascinated scientists around the world.

CDs are oligomeric materials produced by enzymatic degradation of starch via cyclodextrin-glycosyltransferase. These cyclic oligomers are shaped like truncated cones with a hydrophilic outer surface and a relatively lipophilic central cavity. The central cavity enables the complex formation of CDs with guest molecules. CDs consist of six, seven, eight, and a greater number of D-glucose units, joined through α-(1, 4) glycosidic linkages to form rings, and are known as α, β and γ-cyclodextrin consecutively [12,13,14]. β-cyclodextrin (β-CD) possesses the most suitable cavity size for complex formation with many drugs [15] and is the lowest-priced [16], making the cyclodextrin (CD) of commercial interest [17]. However, native CDs have several limitations such as the inability of including certain hydrophilic compounds or high molecular-weight drugs, low aqueous solubility, and toxicity when it is administered intravenously [18,19,20]. As a consequence, specific applications required overcoming the aforementioned limitations by chemical modifications of CD structures [21,22,23]. A common approach to improve the performance of parent CDs is cross-linking [24], producing water-soluble and insoluble cyclodextrin polymers or cyclodextrin-based nanosponges (CD-NSs). CD-NSs are chemically cross-linked polymers that have many attractive features for use as hydrogels. The structure of CD-NSs is strongly dependent on the type of the cross-linker [25]. Dianhydrides are suitable cross-linkers due to their reactivity with nucleophiles, such as the OH groups of CDs structure. The unique property of the NSs synthesized is swelling in the biological and aqueous environment [22,26]. The cross-linking reaction of dianhydrides with OH groups is discussed widely in the literature [22]. Ester-bridged CD-NSs are capable of encapsulating a wide variety of compounds due to the hydrophilic channels of their porous structure and the presence of the lipophilic nanosized cavities of cyclodextrin monomers [27,28]. This behavior has been exploited to improve the solubility, stability, and bioavailability, and to control the release of poorly water-soluble drugs [29,30,31]. High encapsulation efficiency and slow-release kinetics are due to the electrostatic interactions of the carboxylic groups of dianhydride bridges with polar moieties of hydrophilic drugs [32] or due to the inclusion complex formation with lipophilic drugs [33]. The β-CD:cross-linker molar ratio influence on drug release was investigated. It was found that the β-CD and cross-linker affect the nano-channels produced and, therefore, the extent of hydrogel swelling, drug loading capacity, and the rate of drug release [34,35]. Moreover, to control and design the delivery kinetics, deep knowledge of the cross-linking properties of polymeric structures is a mandatory step [36]. Cross-linking density affects the final characteristic properties of CD-NSs [3,37], controlling the swelling of the hydrogel and the consequent mechanical properties, two key properties when ester-bridged CD-NSs are extensively explored as drug delivery systems [38].

Significant advances on the synthesis of CD-NSs [39], and their applications ranging from the environment [40] to other fields such as pharmacy, chemistry, agriculture, gene delivery, biomedicine and biotechnology, food, cosmetics, biocatalysis, etc., [6,7,8,9,10,22] have emerged over years. As judged from the historical development of CD-NSs [39], they have been the subject of numerous surveys, heading towards greener processes such as the CD-NSs synthesis in natural deep eutectic solvents (NADES) [41] and solvent-free CD-NSs synthesis [42]. Studies performed on the polymeric structure of ester-bridged βCD-NSs based on PMDA [27,43,44,45] showed that the crosslinking degree is strongly dependent on the PMDA:β-CD molar ratio used during the CD-NSs synthesis, related to the swelling capacity as an important parameter. Nevertheless, a deep study related to their crosslinking density and the molecular weight between two cross-links points has never been probed. Therefore, this subject is currently of great interest and a very challenging task.

In light of this, the aim of our study was to investigate the effect of the cross-linking density on the swelling and mechanical properties of PMDA:β-CD n:1 molar ratio (n = 2, 3, 4, 5, 6, 7, 8, 9, and 10), using Flory–Rehner theory and rheology. The water absorption capacity (WAC), and the fundamental parameters of polymer network such as molecular weight between cross-links (M_c_), cross-linking density (*υ*), storage modulus (*G’*), and loss modulus (*G’’*) were determined.

This study is a novelty for NSs literature because it is the first time that the above-mentioned techniques have been used to investigate the influence of the cross-linking density on βCD-NSs physicochemical properties.

## 2. Materials and Methods

### 2.1. Materials

β-CD of molecular weight (M_w_) 1134.98 g/mol was kindly provided as a gift by Roquette (Lestrem, France). β-CD was dried in the oven at a defined temperature up to constant weight before its usage, to remove any traces of water. PMDA (97%), dimethylsulfoxide (DMSO, ≥99.9%), triethylamine (Et_3_N, ≥99%), and acetone (≥99% (GC)) were purchased from Sigma-Aldrich (Darmstadt, Germany).

### 2.2. Methods

#### 2.2.1. Synthesis of PMDA:β-CD NSs

The synthesis of PMDA:β-CD NSs was performed by modifying the procedure already mentioned in the existing literature [46], based on the synthetic procedure described in the Italian patent [47]. Nine types of NSs were synthesized by dissolving 4.886 g of anhydrous β-CD in 20 mL of DMSO in a round bottom flask until a transparent uniform mixture was observed. Afterwards, 1.25 mL of Et_3_N was used as a catalyst with the subsequent addition of PMDA as a cross-linker by applying β-CD:PMDA molar ratios of 1:2, 1:3, 1:4, 1:5, 1:6, 1:7, 1:8, 1:9, 1:10 (Table S1 in Supplementary Material). The cross-linking reaction was exothermic and, therefore, was carried out under intense magnetic stirring at room temperature.

**Figure 1:**
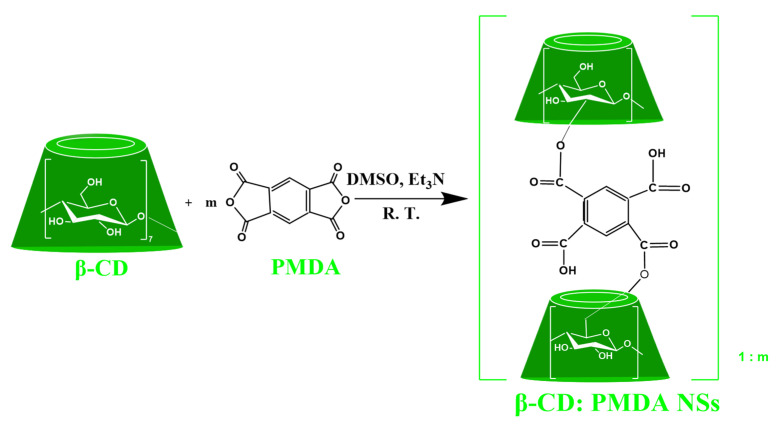
Esterification of β-CD with pyromellitic dianhydride.

The polymerization was completed within a few minutes, especially for higher molar ratios, obtaining a solid with a great yield that was allowed to stand for 24 h. The solidified mass was broken up and manually ground in a mortar. Then, it was stirred with an excess of deionized water, repeatedly, until a clear supernatant solution is obtained. The purification process was speeded up using Buchner filtration system. The unreacted reagents or residual reaction by-products were completely removed in Speed Extractor (BUCHI E-914) with acetone for around 20 min. Finally, the NSs were air-dried, milled, and utilized for characterization as white homogeneous powders. Figure 1 presents the polymerization reaction. A schematic representation of the NSs synthesis is provided in the Supplementary Material (Figure S2).

#### 2.2.2. Swelling Studies

The kinetics of NSs swelling, having various degrees of crosslinking, was studied by following their increase in weight and volume when immersed in water.

**Figure 2:**
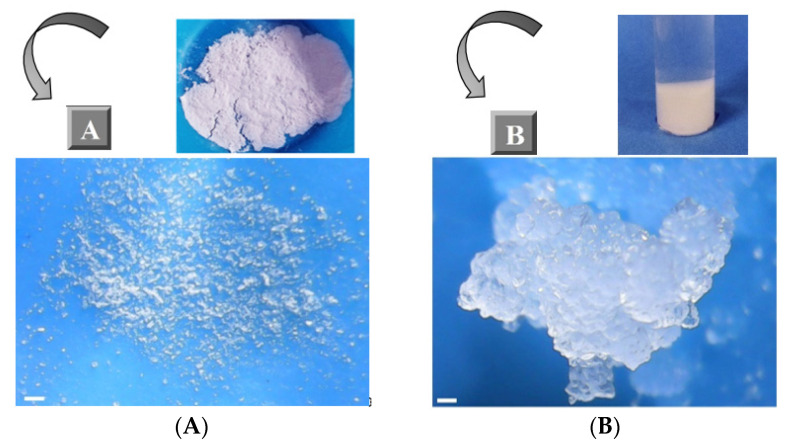
Images of β-CD:PMDA NS with molar ratio 1:6 in a dry state (**A**) and a swollen state (**B**). Scale bar: 1 mm. The images of other molar ratios are presented in Supplementary Material (Figure S3).

The swelling measurements were performed by immersing 500 mg of dry powder (200 mg in the case of molar ratio 1:2), in deionized water (in-12 mL test tubes filled up to 10 mL), and blending them, in the beginning, using a Vortex Mixer. The test tubes were sealed and maintained at room temperature. After 0.5, 2, 4, 6, 12, 24, 48, and 72 h the mixtures were centrifuged to obtain a layer of water-bound material and free unabsorbed water. After removing the supernatant, the residual amount of free water was blotted off using tissue paper and the weight was recorded. The used water was replaced with fresh deionized water after each recording. The dry (Figure 2A) and swollen NS samples (Figure 2B) were observed with an optical microscope equipped with a photo-camera.

All swelling measurements were performed in triplicate for each molar ratio and the data obtained were expressed as mean values ± SD.

The swelling rate in percentage (%S) or the water absorption capacity (%WAC) was calculated using the equation [48,49,50,51]:

(1) $$\mathrm{WAC}\;\left(\%\right)=\frac{m_{t}-m_{o}}{m_{o}}\times 100$$

where *m_t_* is the weight of the swollen sample at time *t* and *m_o_* is the initial weight of the dry sample.

#### 2.2.3. Cross-Linking Density Determination Using Swelling Experiments

##### Flory–Rehner Theory

A weighed amount of about 500 mg of β-CD:PMDA (200 mg in the case of the NS with β-CD:PMDA molar ratio of 1:2) was dispersed in 10 mL of deionized water in a 10-mL test tube and allowed to swell for two hours. The swelling study permitted the calculation of the polymer volume fraction in the equilibrium-swollen polymer (*υ_2m_*) that is used to calculate the cross-linking density (*υ*) using the Flory–Rehner theory. The number of cross-links per unit volume in a polymer network is defined as cross-linking density [37]. The polymer volume fraction, an important parameter used for the characterization of the polymer network structure, is related to the quantity of water that a polymer can incorporate. It is expressed as a ratio of the volume of polymer (*V_p_*) to the volume of the swollen polymer or gel (*V_gel_*) at equilibrium [52,53].

(2) $$\upsilon_{2m}\;=\;\frac{Vp}{Vg}\;=\;\frac{V_{g}-V_{w}}{V_{g}}\;=\;1\;-\;\frac{m_{w\;}\rho_{g}}{\rho_{w}m_{g}}$$

*ρ_g_* is the density of the swollen polymer, *ρ_w_* is the density of water, *m_g_* is the mass of the swollen polymer at equilibrium and *m_w_* is the mass of water present in the swollen polymer. The densities of both dry and swollen polymers were determined using a pycnometer (Figure S4 in Supplementary Material). The details are presented in Supplementary Material (Sections S4 and S5). The experimental values of both densities are presented in Table S2 (Supplementary Material).

The M_c_ is calculated using the Flory-Rehner equation (M_c_) [54,55]:

(3) Mc =V1[(υ2m)^1/3 −(2fυ2m)]−[ln(1−υ2m) +υ2m + χ1 (υ2m)2] 

where *χ*_1_ is the Flory–Huggins solvent-polymer interaction parameter, *V*_1_ is the molar volume of water as a swelling agent and f is the functionality of the cross-links. The relationship between M_c_ and *υ* is given by the following equation:

(4) $$M_{c}=\frac{\rho_{p}}{\upsilon }$$

where *ρ_p_* is the polymer density.

The procedure of the *χ*_1_ determination is published elsewhere [26,56], but there is no literature value for PMDA CD polymers, therefore, in this work, the literature value of *χ*_1_ = 0.473 for dextran was employed. Functionality (*f*) is the maximum number of chemically linked polymer chains at a cross-link. For the β-CD:PMDA system *f* = 3 was taken into consideration, based on the three hydroxyl groups of the glucose unit, and *V*_1_ = 18 cm^3^ mol^−1^. All the measurements were performed in triplicate for each molar ratio and the data obtained were expressed as mean values ± SD.

##### Rheological Measurements

Rheological measurements were performed in a Rheometer TA Instruments Discovery HR 1 by following the procedure described in the literature with some modifications [57,58]. The instrument was equipped with 20 mm diameter stainless steel plate geometry and Peltier plate temperature control. Frequency sweep measurement was performed from 100 to 0.2 rad/s and stress amplitude of 2%. The amplitude sweep test was used to check the value of stress amplitude, guaranteeing the performance of the measurements within the linear viscoelastic region. The oscillatory shear mode was used to determine the shear modulus (G), in particular the storage modulus (G’) and the loss modulus (G’’) of the swollen NSs as a function of frequency (Frequency Sweep test) and as a function of shear strain (Amplitude Sweep test). G’ and the G’’ are two significant parameters for the characterization of viscoelastic materials. The sample was placed between the upper parallel plate and stationary surface with varying gap size (1 mm and 2 mm) adapted following the same procedure previously detailed. The loading procedure was a practical difficulty because care must be taken to avoid the formation of air bubbles. As the wall-slip formation is inevitable [59], to overcome it, a roughened surface geometry, such as a crosshatched plate, was employed to improve the contact between the geometry and the sample. After sample loading, the sample edge was carefully trimmed with a spatula to maintain the proper surface shape during the measurements and to avoid errors (Figures S5 and S6 in Supplementary Material). However, the effects of overfilling and solvent trap on rheological measurements were also studied (Figure S7 in Supplementary Material). Samples were equilibrated for 5 min, before the experiments, to allow the relaxation of the whole structure. A delay of 5 min was applied to measure the initial structure level of the samples before shearing and to eliminate any disturbance created by the measuring geometry [60]. The temperature at 25 °C was controlled by a water bath circulator. To ensure the reproducible state of the samples, the measurements, for each molar ratio, were accomplished in triplicate recording their average. The data obtained were expressed as mean values ± SD.

The fraction of elastically effective network chains is determined by the modulus measurements. The theory of elasticity, developed by Flory, predicts the equilibrium shear elasticity. The value of the plateau modulus *G’_p_*, obtained by rheological measurements, is directly related to the number of elastically effective chains per unit volume (*υ_e_*) as expressed by the following equation:

(5) $$G_{p}^{\prime }=\left(1-\frac{2}{f}\right)\times \upsilon_{e}\times \mathrm{RT}$$

*υ_e_* is the molar number of elastically effective network chains per unit volume estimated in mol m^−3^, R is the universal gas constant (8.314 J mol^−1^ K^−1^), T is the temperature, *f* is the functionality formerly defined [61].

## 3. Results and Discussions

### 3.1. Synthesis of Ester-Bridged βCD-NSs Based on PMDA

NSs of different cross-linking density were successfully synthesized by the esterification of the hydroxyl groups of β-CD with PMDA, used as a crosslinker. The NSs were prepared with stoichiometric ratios 0.285, 0.428, 0.571, 0.714, 0.857, 1, 1.142, 1.285, and 1.428 mol of PMDA per mole of glucose unit. β-CD esters are synthesized by the ring-opening reactions of PMDA. The ring-opening of anhydride is caused by the reaction of the hydroxyl groups in β-CD structure with PMDA, via nucleophilic attack, using Et_3_N as a catalyst. This results in the formation of carboxyl and ester groups in the polymer network. The four carbonyl groups attached to one benzene ring in a coplanar conformation, typical of PMDA structure, show a high tendency to accept an electron [62]. Thus, PMDA is considered the most reactive dianhydride monomer. Ester-bridged βCD-NSs based on PMDA contain free carboxylic acid groups, therefore they can form complexes with both apolar organic molecules and cations. Depending on the amount of PMDA added, products with a variable number of acid groups and different hardness were obtained. By varying the ratio of the cross-linker used in the reaction, there was observed a change in the physical appearance as well as the yield of the product. The network chain between the cross-links becomes shorter and entangled, as the amount of PMDA increases, therefore, the polymer is strongly interconnected or more rigid. Contrarily, the NSs with less amount of PMDA have low rigidity because the polymer chains are loosely bonded by weak Van der Waals forces or less strongly interconnected, and move easily. The NSs with higher molar ratios resulted in higher yields than the others (Table 1).

### 3.2. Water Absorption Capacity (WAC)

**Figure 3:**
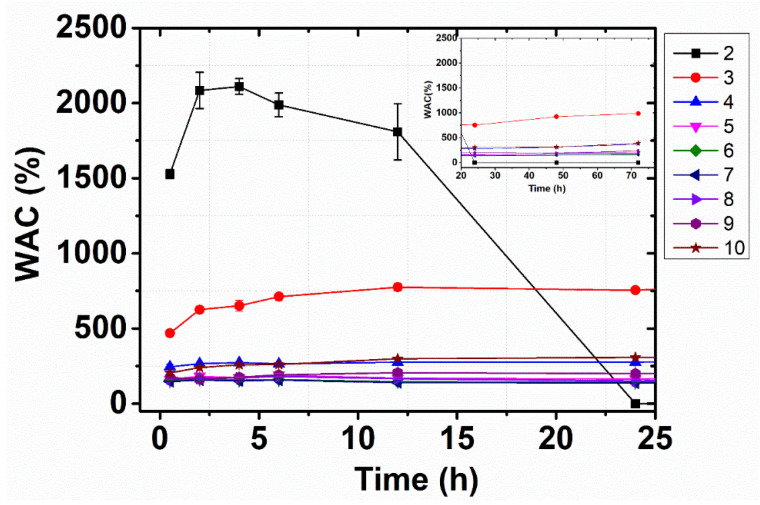
Water absorption capacity (WAC) as a function of the swelling time for each monomer ratio of β-CD:PMDA NSs. The points are the average of three experiments and the bars represent the standard deviation.

**Figure 4:**
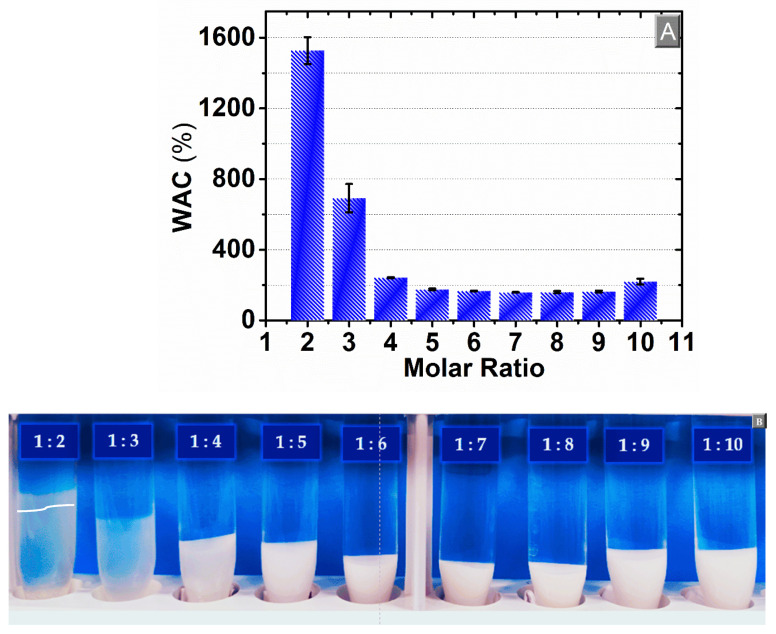
(**A**) Water absorption capacity (WAC) and (**B**) image of β-CD:PMDA NSs in a swollen state as a function of cross-linker to monomer ratio of β-CD:PMDA NSs. The points (**A**) are the average of three experiments and the bars represent the standard deviation.

As the network of the β-CD:PMDA NSs bears hydroxyl and carboxylic acid groups, there is a high affinity for water molecules. The capacity to absorb water as a function of time and ratio of cross-linker to monomer was determined. From Figure 3 it can be seen that the maximum water absorbency was achieved after a few hours, followed by plateauing of the absorption up to 72 h for most of the samples. Besides, the water absorption capacity of the β-CD:PMDA NS with the lowest level of cross-linking (1:2) was gradually reduced over time and the gel hydrolyzed after 24 h, as a consequence of the polymer network degradation. The effect of the content of cross-linker on water absorption capacity is shown in Figure 4A. The values of water absorption capacity decrease as the content of cross-linker are increased between the molar ratio 1:2 (1526%) and 1:5 (174%), as detailed in the Supplementary Material (Table S3a). Above, the water absorption capacity remains almost constant (1:6, 1:7, 1:8, 1:9) and then it rises in the case of 65% cross-linker (molar ratio 1:10). Table S3b,c in Supplementary Material present the experimental values of WAC as the function of the swelling time.

In the case of 27% cross-linker (1:2), as presented in Figure 3 and Figure 4A, can be observed that is challenging to determine a physically meaningful value of WAC. The image in Figure 4B shows that the opacity of the gel increases with the content of PMDA. This substantiates previous findings in the literature demonstrating that the gels of the higher concentration region of the cross-linker are opaque [63]. Opaque gels are characterized by a heterogeneous network structure where particle aggregates are sufficiently large to scatter light [64]. This heterogeneity can be explained by the fact that the increase of cross-links restricts the movement of polymer chains [48,50], leads to the compaction of the structure, and hampers the diffusion of water in the polymer network [65]. This results in a decreased degree of swelling of gel. The driving force, in the swelling of cross-linked polymers, is due to the contribution of normal entropy and enthalpy changes associated with the mixing of solvent and solute molecules (Figure S1 in Supplementary Material). Further, changes in configurational entropy result from the dilution of flexible chain molecules. The dispersion tendency of cross-linked polymers is opposed by a decreased configurational entropy of the polymer chains held between cross-links. This is caused by an elastic restrain force. As the network expands, these chains are forced to assume more elongated, less probable configurations, therefore, the swelling capacity is lower at higher crosslink ratio [54,66,67].

### 3.3. Flory–Rehner Theory

Numerous theories have been proposed to predict network structures. Flory and Rehner convincingly, through their mathematical and conceptually simple theory, interpret the swelling of polymeric networks [53]. The β-CD:PMDA NSs are hydrophilic polymers containing carboxylic groups, as detailed in the previous section, in equilibrium with the carboxylate groups in the presence of water. As a consequence of the electrostatic repulsion of negative charges, the polymer chains are expanded [68]. Besides, the swelling comes as a result of the cross-links presence within a polymer network that prevents the dissolution of the polymer. If the network structure is not dissolved in water but only swells, a state of equilibrium swelling can be reached, as explained by the Flory–Rehner theory. According to this theory, the free energy change ΔF involved in the mixing of polymer with the solvent consists of ordinary free energy of mixing ΔF_M_, and the elastic free energy ΔF_el_.

(6) $$\Delta F=\Delta F_{M}+\Delta F_{\mathrm{el}}$$

The mixing free energy (ΔF_M_) is a function of the polymer volume fraction (*υ_2,m_*) and the Flory–Huggins solvent-polymer interaction parameter (*χ*). Its derivative, with respect to the number of solvent molecules (*n*_1_), can be written as:

(7) $$\frac{\partial \Delta F_{M}}{\partial n_{1}}=\mathrm{RT}\times \left\{ln(1-\upsilon_{2,m})+\upsilon_{2,m}+\chi \upsilon_{2,m}^{2}\right\}$$

As β-CD:PMDA NSs are highly cross-linked systems, the contribution from the configurational entropy of the network during the swelling is considered. According to the aforementioned, the elastic free energy ΔF_el_, associated with the expansion of the polymer network, is equal to −TΔS_el_ representing the entropy change associated with the network configuration change. Therefore, ΔF_el_ derivative can be expressed as:

(8) ∂ΔFel∂n1=RTυe×V1V0×[(υ2,m)^1/3−υ2, m2],  υe V0=ρMc

Combining both contributions, from Flory–Huggins (Equation (7)) and the configurational entropy of the network (Equation (8)), the overall free energy can be computed as:

(9) ΔF=RT×[ln(1−υ2,m)]+υ2,m+χυ2,m2+ρV1Mc×[(υ2,m)^1/3−υ2,m2]

When the equilibrium is reached, ΔF = 0, therefore, Equation (9) can be rearranged as [26,38,54,55,56,69]:

(10) −[ln(1−υ2,m)]+υ2,m+χυ2,m2=ρV1Mc×[(υ2,m)^1/3−υ2,m2]

The details of the Flory–Rehner theory are described in the Appendix A.

**Figure 5:**
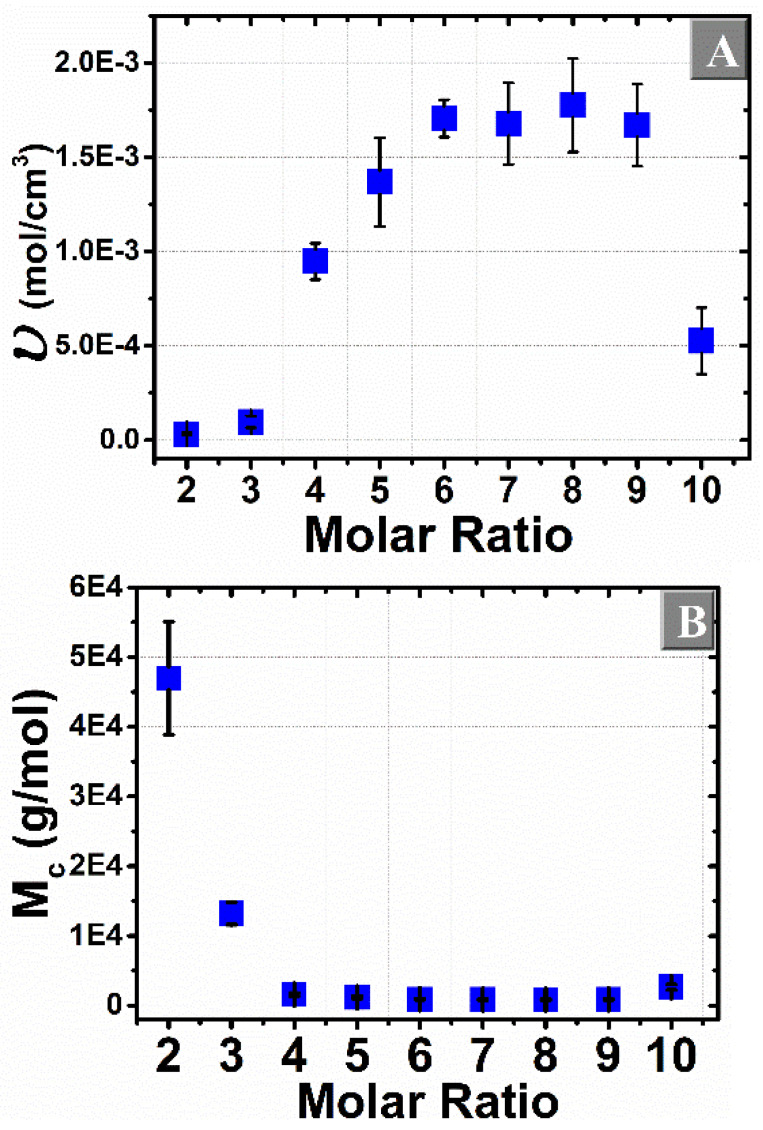
Mean values of (**A**) cross-linking density υ (mol/cm^3^) and (**B**) molecular weight between cross-links M_c_ (g/mol) from the equilibrium swelling of prepared NSs; vertical bars represent the standard deviation.

The swelling of a polymer gel leads to a decrease in chain configurational entropy and an increase in entropy of mixing of solvent with the polymer. The absorption of water is a consequence of osmotic forces, whereas the decrease in entropy is caused by the stretching of the polymer chains and it gives rise to the elastic force of the polymer network. The equilibrium is reached when the opposing entropies are balanced, as extensively studied in the literature [67,70,71,72]. According to the Flory–Rehner theory, the attraction of the water molecules by the hydrophilic polymer chains is described by the mixing energy, specifically the first three terms of Equation (10) *ln*(1−*υ_2,m_*), *υ_2,m_* and *χυ^2^_2,m_* [73]. Meanwhile, the last term [*V_1_ ×* (*υ_e/_V_0_*) × (*υ*_2_^1/3^ − *υ*_2_/2)] describes the elastic free energy associated with the stretching of the gel [74,75]. The water absorption or swelling capacity of the polymer network depends on the degree of cross-linking. Therefore, swelling tests were performed on the β-CD:PMDA NSs and the Flory–Rehner equation given previously (Equations (3) and (4)) was applied to determine the degree of cross-linking of each molar ratio. Figure 5A shows that by increasing the molar ratio up to 1:6 the cross-linking density increases, reaching a “plateau” (1:7, 1:8, 1:9) and then it decreases in the case of molar ratio (1:10), following a Gaussian distribution. This is in agreement with a previous study by Rossi et al. [27]. According to this study, the increase in NS stiffness with the increasing of the cross-linker amount reaches a maximum at a certain limit (1:6). If the molar ratio cross-linker to β-CD is higher than 6, it leads to branching rather than further cross-linking of the CD network. The maximum degree of cross-linking, probably due to steric effects, has already been observed by the combined use of Raman and infrared spectroscopy supported by quantum chemical calculations [44], as well as inelastic light-scattering experiments [27]. As expected, at a higher degree of crosslinking Figure 5B), the average distance between two cross-link points (M_c_) becomes shorter and the network structure becomes denser. Thus, the experimental values of M_c_ increase with the decreasing of the cross-linking ratio in β-CD:PMDA NSs.

The experimental values of molecular weight between cross-links (M_c_), cross-linking density (*υ*), and polymer volume fraction (υ_2m_) are shown in Table S4 provided in the Supplementary Material. The molecular weight between cross-links (M_c_) is the parameter that describes the basic structure of the gel and it relates to the ability of the polymer network to swell. An increase of molecular weight between crosslinks (M_c_) is accompanied by a decrease of cross-linking density, followed by a decrease of swelling ratio. High M_c_ values correspond to a loosely cross-linked network and increased swelling ratio. Therefore, the cross-linked structure of the NSs matrix can be controlled effectively by adjusting the amount of cross-linking agent used in the synthesis process.

### 3.4. Rheological Measurements

**Figure 6:**
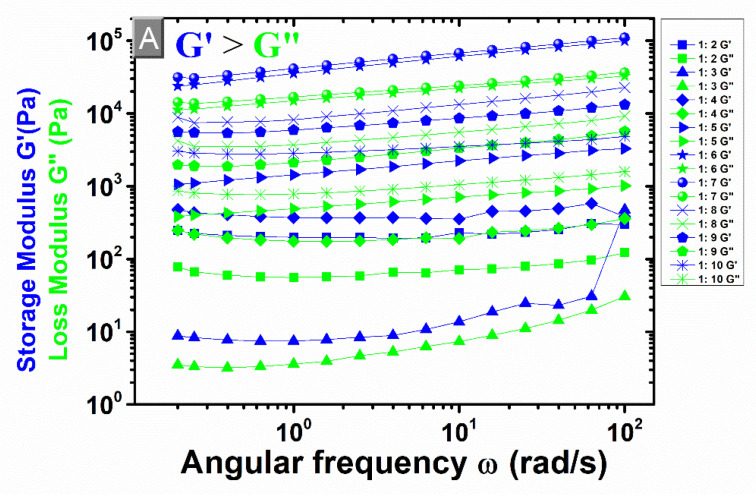

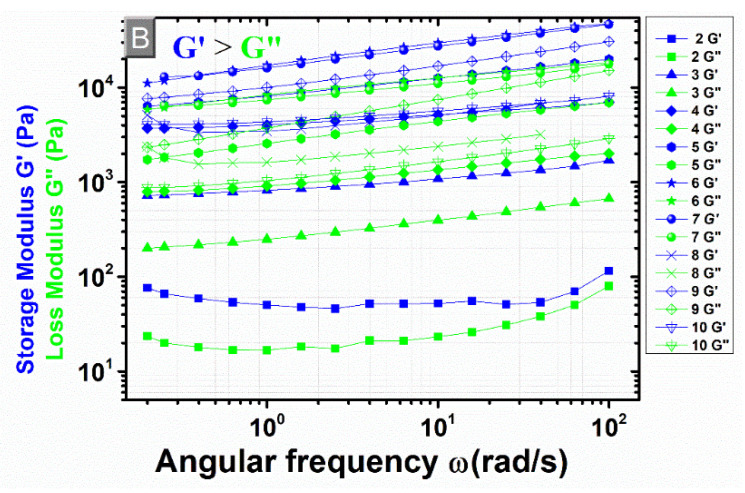
Storage (G’) and loss (G’’) modulus versus angular frequency for β-CD:PMDA molar ratio of 1:3, 1:4, 1:5, 1:6, 1:7, 1:8, 1:9, 1:10. (**A**) 1 mm gap size; (**B**) 2 mm gap size.

**Figure 7:**
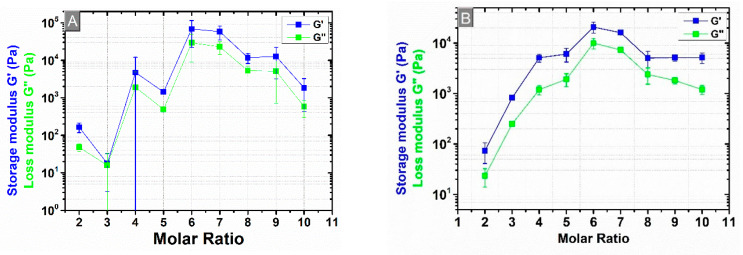
Storage (G’) and loss (G’’) modulus versus molar ratio of β-CD:PMDA (1:3, 1:4, 1:5, 1:6, 1:7, 1:8, 1:9, 1:10) at an angular frequency (ω) of 1 rad/s; (**A**) 1 mm gap size; (**B**) 2 mm gap size.

The swelling capacity is also a crucial property to determine the mechanical stiffnesses of β-CD:PMDA NSs. Rheology is a technique frequently used to investigate the mechanical properties of polymer networks. It studies the deformation and flow of material, by applying a force called shear stress or a deformation called a strain, using a rheometer. The rheometer consists of two basic components separated by the sample. The viscoelastic behavior will appear as a response of the material to the applied force [76,77,78,79]. A stress sweep test on a parallel plate rheometer under controlled strain conditions was performed to study β-CD:PMDA NSs morphology. Shear modulus (G), in particular, the storage modulus (G’) and the loss modulus (G’’) are significant parameters which enable the characterization of viscoelastic materials. The measure of the deformation energy stored by the sample during the shear process is called G’ whereas the consumption of the deformation energy by the sample during the shear process is called G’’. Energy storage materials are characterized by reversible deformation behavior because of the unchanged shape after a load cycle. The viscous behavior of test material is represented by G’’ whereas the elastic ones by G’. Therefore, the material appears as a gel when G’’ is lower than the G’ and as a liquid when G’ is lower than G’’. The cross-point expresses the oscillation stress at which G’’ and G’ are equal [57,69,76,80,81,82]. Figures S8 and S9 in Supplementary Material present the effect of overfilling and solvent trap on rheological measurements, with a gap of 1 mm. Figure S8 in Supplementary Material shows that the viscoelastic properties of the synthesized NSs are a strong function of the test frequency. An abrupt increase in G’ and G’’ was observed with increasing angular frequencies for NSs at molar ratio of 1:5, 1:6, 1:7, 1:8, implying that the samples still maintain their strong gel and elastic characteristics. Therefore, the gel consisting of the solvent immobilized within a three-dimensional polymer network represented an elastic solid. Whereas, for NSs at molar ratio of 1:3, 1:4, 1:9, and 1:10, the curve indicates a plateau towards the highest angular frequencies (plateau modulus, *G_p_’*). Exemplary results in Figure S9 in Supplementary Material, determined for β-CD:PMDA molar ratio of 1:3, 1:4, 1:5, 1:6, 1:7, 1:8, 1:9, 1:10, at an angular frequency (ω) of 1 rad/s, demonstrated that G’ is higher than G’’ for all molar ratios, confirming once more the gel-state behavior of the NSs. Moreover, an increase of both the G’ and the G’’ is more pronounced as the PMDA content increases up to molar ratio 1:5 and 1:6. The further increase of the cross-linker content (molar ratios 1:7, 1:8, 1:9, and 1:10) results in a drastic decline of G’ and G’’. Besides, the lowest molar ratio of NSs such as 1:2 is inclined towards the liquid-state behavior. Therefore, its thickness was not enough for the gap showing the poorest mechanical properties due to its highest swelling degree. This confirmed what one study already observed [77], a decrease in the mechanical properties of the hydrogels, and an increase of the water absorption capacity are caused by the arrangement of water molecules inside the hydrogel structure. The presence of water molecules affects the rheological properties of the hydrogels, making the hydrogel softer under mechanical stress and vice versa. From the literature [83], the removal of the extra sample outside the geometry is considered an essential step in experimental rheology to minimize the experimental errors. Figure 6 and Figure 7 present the rheological measurements with varying gap of 1 mm (Figure 6A and Figure 7A) and 2 mm (Figure 6B and Figure 7B), trimming the sample before the experiment and not using the solvent trap. The lowest molar ratio of NSs such as 1:2 is also displayed among other results. The results show approximately the same dependency of G’ and G’ from the PMDA content as previously described for Figures S8 and S9 (Supplementary Material). As expected, the way of sample loading and gap size varying can cause errors in final results. In most of the molar ratios, the G’ and G” are decreased as the gap height is increased from 1 mm to 2 mm. Further, the errors are higher at 1 mm gap size (Figure 7A) than at 2 mm gap size (Figure 7B).

At low frequency, the storage modulus (G’) tends toward a plateau that defines the cross-linking density of the network. Therefore, the influence of the cross-linker ratio on G’ and G’’, with gap size varying (1 mm and 2 mm), is studied. Generally, when the measured values of G’’ were observed to be on the order of 0.1% to 5% of the G’ values, the inclusion of G’’ in calculations could be neglected [61]. Hence, G’ is used to calculate the number of effective chains per unit volume (*υ_e_*) according to Equation (5).

**Figure 8:**
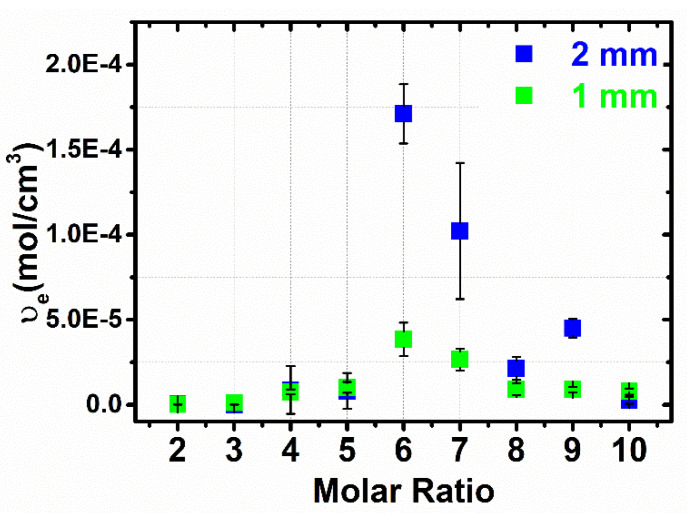
Effective sub-chain density (moles of effective sub-chains per unit volume) as a function of added cross-linker content. 1 mm; 2 mm; gap sizes, removing the extra sample outside the geometry and without solvent trap.

The chains are considered elastically effective when are connected at both ends to cross-links which are further defined as junctions with three or more paths to the gel network [84]. Figure 8 shows that by increasing the cross-linker content at a certain amount such as the molar ratio 1:6 (1 mm gap size) and 1:6 (2 mm gap size), the *υ_e_*increases as well. A higher cross-linker concentration should promote a more efficient cross-linked network with higher gel strength. It is observed a decrease of *υ_e_* by further increasing the molar ratio, and the errors are higher at 1 mm gap size than at 2 mm gap size. The trend of an increase in the *υ_e_* with increasing the cross-linker content at a certain amount (1:6 molar ratio) is observed in Figure S10 (Supplementary Material). It presents the *υ_e_*calculated from rheological measurements carried out in the presence of overfilling and solvent trap. Therefore, the values of *υ_e_,* compare to abovementioned, in these conditions are higher (Figure S11 in Supplementary Material), adding errors to the data. This can be rationalized with what is already investigated in literature [83] that overfilling and gap size cause data errors. To sum up, with the increasing of the cross-linker amount, the G’ values increase (Figure 7A,B) because of the increment of *υ_e_* points (Figure 8). The effectiveness of the cross-linking agent can be associated with the occurrence of inhomogeneities within the network [85]. The experimental values of G’, G” and *υ_e_*, at 1 mm and 2 mm gap size, are presented in Tables S5–S8 of the Supplementary Material.

### 3.5. Comparison of the Cross-Linking Density Determination Based on Two Different Methods

**Figure 9:**
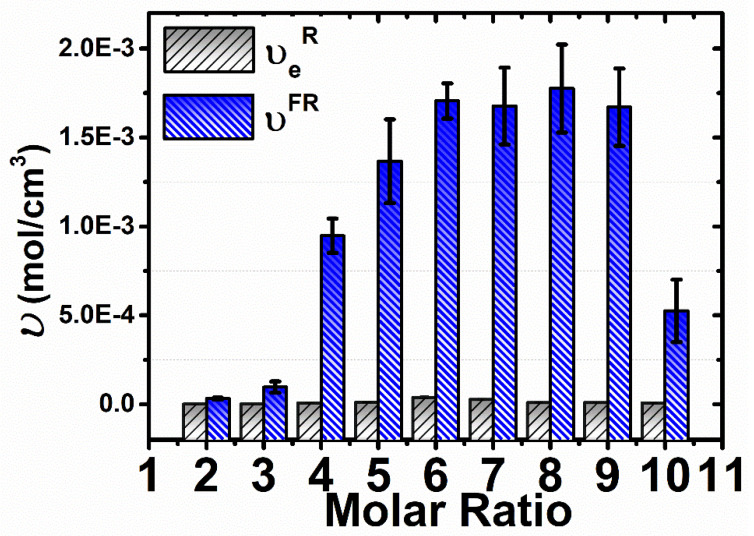
Comparison of the cross-linking densities obtained by two different methods: Flory–Rehner theory (*υ^FR^*) and rheological measurements(*υ_e_^R^*) for β-CD:PMDA NSs, at 2 mm gap size.

The profiles of cross-linking density vs. molar ratio obtained by both the Flory–Rehner and the rheological methods are in close agreement with each other. In both cases, a maximum cross-linking density appears in the range of 6–8 PMDA*:*β-CD molar ratio. However, Figure 9 shows that the values based on the Flory–Rehner equation are higher than those based on the rheological measurements. This trend of higher values in the case of Flory–Rehner theory compared to other methods has also been observed in other studies focused on the evaluation of the cross-linking density of rubbers [86] and hydrogels based on cellulose [56]. Returning to Equations (3)–(5), it can be observed that the cross-linking density determination is treated differently. According to Equations (3) and (4), both the mixing and elastic contribution are considered. Thus, all monomer units belonging to the polymer network contribute to the calculation of *υ^FR^*. The higher values resulted from the Flory–Rehner equation are attributed to its numerous parameters, obtained by various independent methods, affecting the final values. Whereas, Equation (5) considers just a certain fraction of the polymer network to calculate *υ_e_* [55]. Therefore, the lower values of *υ_e_*, obtained from the rheological measurements, display probably the presence of chain ends but not of the entanglements. This may be as a consequence of the number of added effective sub-chains calculation from the number of added cross-links. Taken together, this highlights the assumption that two effective sub-chains are equally with each added cross-link [87]. At this stage it is not possible to fully explain the gap between the values of cross-linking density, as it may arise both from the experimental procedure used to prepare the gels and from the intrinsic differences between the two models applied. Future studies are necessary to reduce such gap, starting from the determination of the interaction parameter *χ* and its dependence on the content of PMDA, as well as the application of different models able to take into account the entanglement points of the polymer network.

## 4. Conclusions

In this study, chemically cross-linked β-CD-based NSs were successfully synthesized varying the relative amount of the PMDA as a cross-linker (in stoichiometric proportions of 2, 3, 4, 5, 6, 7, 8, 9, and 10). Based on the results the following particular features of β-CD-based NSs can be concluded: (a) the capability to absorb a huge amount of water concerning their weight or noted as swelling capacity, due to the presence of -OH and -COOH pendant groups, and (b) the control of the cross-linking density and rheological characteristics in a stoichiometric way. The water absorption or swelling capacity, measured as a function of the cross-linker amount present in β-CD:PMDA NSs showed a maximum of up to 1526 g H_2_O/g dry sample. The water absorption capacity was lower when the concentration of PMDA increased. Both Flory–Rehner theory and rheology, through equilibrium swelling experiments, have showed that the final β-CD-based NSs network exhibited a cross-linking density distribution. These models yielded a reasonably good fit of the data and good agreement between the theory and experiment. The cross-linking density values reached a maximum with increasing the cross-linker content at a certain amount (between 1:6 and 1:7, β-CD:PMDA). Additionally, the M_c_, calculated by Flory–Rehner theory, decreased with increasing cross-linking ratio. Rheology was used to study the correlations between the cross-linking determination and mechanical properties of the NSs network. The gel-state behavior of the NSs is confirmed by higher values of G’ than G’’ for all angular frequencies, and both G’ and G’’ were dependent on PMDA content. As PMDA increased at a certain amount, higher G’ and G’’ were pronounced.

These findings have a huge impact on a wide variety of practical uses, especially for pharmaceutical (Figure S12 in Supplementary Material) and biomedical purposes. Understanding the correlation between the structural features of PMDA βCD-based NSs and their physicochemical properties will allow one day to identify rapidly and effectively the right synthesis method, in terms of monomers formulation and reaction condition, to fulfill the requirements of the desired specific applications.

## Figures and Tables

**Table 1:** The yields of the final products with different amounts of the cross-linker.

Molar Ratios (β-CD:PMDA)	Yield (%)
1:2	56%
1:3	74%
1:4	>95%
1:5	>95%
1:6	>95%
1:7	>95%
1:8	>95%
1:9	>95%
1:10	81%

## Data Availability

The data presented in this study are available on request from the corresponding author.

## Supplementary Materials

The following are available online at https://www.mdpi.com/1996-1944/14/3/478/s1. Figure S1: Chemical modification of the polymer; Figure S2: Scheme of the synthesis of β-CD:PMDA NSs; Figure S3: Images of β-CD:PMDA NS molar ratio in a dry state (A) and in a swollen state (B); Figure S4: Pycnometer; Figure S5: Sample loading in a rheometer, 1 mm gap size, removal of the extra sample outside the geometry and of the solvent trap; Figure S6: Sample loading in a rheometer, 2 mm gap size, removal of the extra sample outside the geometry and of the solvent trap; Figure S7: Sample loading in a rheometer, 1 mm gap size. Non-removal of the extra sample outside the geometry and of the solvent trap; Figure S8: Storage (G’) and loss (G’’) modulus versus angular frequency for β-CD:PMDA molar ratio of 1:3, 1:4, 1:5, 1:6, 1:7, 1:8, 1:9, 1:10. 1 mm gap size without removing the extra sample outside the geometry and with solvent trap; Figure S9: Storage (G’) and loss (G’’) modulus versus molar ratio of β-CD:PMDA (1:3, 1:4, 1:5, 1:6, 1:7, 1:8, 1:9, 1:10) at an angular frequency (ω) of 1 rad/s; 1 mm gap size without removing the extra sample outside the geometry and with solvent trap; Figure S10: Effective sub-chain density (moles of effective sub-chains per unit volume, υ_e_, mol/cm^3^) as a function of added cross-linker content. 1 mm gap size without removing the extra sample outside the geometry and with solvent trap; Figure S11: Effective sub-chain density (moles of effective sub-chains per unit volume, υ_e_, mol/cm^3^) as a function of added cross-linker content, for different rheological procedures as previously described (1 mm a), 1 mm b), 2 mm); Figure S12: β-CD: PMDA NSs as delivery systems: acetyl salicylic acid, imiquimod, lansoprazole, insulin, curcumin, resveratrol, meloxicam, rosuvastatin, rilpivrine; Table S1: The varying amounts of PMDA as a cross-linker in the synthesis of β-CD NSs; Table S2: Calculated densities of both, the gel and powder of β-CD: PMDA NSs having the various amount of PMDA. Mean Values ± SD; Table S3: a.) WAC experimental values of β-CD:PMDA NSs; b.), c.) Water absorption capacity (WAC) as the function of the swelling time to monomer ratio of β-CD:PMDA NSs; Table S4: Calculated physicochemical terms (M_c_, *υ*, *υ_2m_*) of β-CD:PMDA NSs having the various amount of PMDA. Mean Values ± SD; Table S5: Calculated rheological parameters of β-CD:PMDA NSs having various amount of PMDA. Mean Values ± SD. Gap size (1 mm) without removing the extra sample outside the geometry and with solvent trap; Table S6: Calculated rheological parameters of β-CD:PMDA NSs having various amount of PMDA. Mean Values ± SD. Gap size (1 mm) removing the extra sample outside the geometry and without solvent trap; Table S7: Calculated rheological parameters of β-CD:PMDA NSs having various amount of PMDA. Mean Values ± SD. Gap size (2 mm) removing the extra sample outside the geometry and without solvent trap; Table S8: Calculated physicochemical term (*υ_e_*) of β-CD:PMDA NSs molar ratio for different rheological procedures as previously described.

## Appendix A

### Derivation of Equilibrium Swelling Equation

Equilibrium swelling of a gel is calculated by Flory–Rehner theory, by balancing the mixing and elastic contributions to the osmotic pressure [88]. The ordinary free energy of mixing ΔF_M_ and the elastic free energy ΔF_el_ are two terms in which the free energy change ΔF, involved in the mixing of an amorphous polymeric network with a pure solvent, consists. Thus, it can be expressed:

(A1) $$\Delta F=\Delta F_{M}+\Delta F_{\mathrm{el}}$$

The external arrangement of the molecules and their segments enables the computing of the configurational entropy (Δ$S_{M}^{*}$) representing the total entropy change ΔS_M_ on mixing. The Δ$S_{M}^{*}$ combined with the heat of mixing (ΔH_M_) result in the free energy of mixing that is described in terms of the number of solvent molecules (n_1_), the volume fractions of solvent (*υ*_1__)_ and polymer (*υ*_2_) in the swollen state, Boltzman’s constant (k), absolute (T) and the Flory–Huggins interaction parameter (*χ*_1_) as:

(A2) $$\Delta S_{M^{*}}=-k\times \left(n_{1}\times ln\upsilon_{1}+n_{2}\times ln\upsilon_{2}\right)$$

(A2’) $$\Delta H_{M}=\mathrm{kT}\times \chi_{1}n_{1}\upsilon_{2}$$

(A2’’) $$\Delta F_{M}=\Delta H_{M}-T\Delta S_{M}^{*}=\mathrm{kT}\times \left[n_{1}\times ln\upsilon_{1}+n_{2}\times ln\upsilon_{2}+\chi_{1}n_{1}\upsilon_{2}\right]$$

Due to the absence of individual polymer molecules in the network structure, the number of polymer molecules (n_2_) is equal to zero.

$$n_{2}\ln\upsilon_{2}=0$$

Therefore, the free energy of solvent-polymer network mixing is

(A2’’’) $$\Delta F_{M}=\mathrm{kT}\times [\left(n_{1}\ln\upsilon_{1})+\left(\chi_{1}n_{1}\upsilon_{2}\right)\right]$$

The derivative of ΔF_M_ to n_1_, yields:

(A3) $$\frac{\partial \Delta F_{M}}{\partial n_{1}\;}=\mathrm{kT}\times \left[ln\left(1-\upsilon_{2}\right)+\upsilon_{2}+\chi_{1}{\left(\upsilon_{2}\right)}^{2}\right]$$

The elastic-free energy ΔF_el_, associated with the expansion of the polymer network, is equal with −TΔS_el_ representing the entropy change with the change in network configuration. The pressure acting on the swollen gel is defined as the elastic reaction of the network structure. This pressure is sufficient to increase the chemical potential of the solvent in the solution causing the equilibrium state. A state of equilibrium swelling is reached when the chemical potential of the solvent in the solution is equal to the chemical potential of the excess solvent surrounding the swollen gel.

(A4) $$\Delta S=-\frac{\mathrm{kT}\upsilon_{e}}{2}\times \;\left[a_{x}^{2}+a_{y}^{2}+a_{z}^{2}–3–ln\left(a_{x}a_{y}a_{z}\right)\right]$$

This equality is because the internal energy of the network structure does not experience any change from the occurrence of the deformation process during swelling. α_x_ = α_y_ = α_z_ are contributions to deformation from a relaxed state and can be written in terms of the linear deformation factor α_s_ (α_x_ = α_y_ = α_z_ = α_s_) because of the isotropic swelling. It is noted that α_s_^3^ = *V/V_0_* where *V_0_* is the volume of the unswollen polymer and *V* the volume of the swollen gel. Accordingly, *V_0_/V* = *υ*_2_.

According to the aforementioned, the elastic-free energy ΔF_el_ is presented as

(A5) $$\Delta F_{\mathrm{el}}=\frac{\mathrm{kT}\upsilon_{e}}{2}\times \left[3\alpha_{s}^{2}\;-3-ln\alpha_{s}^{3}\;\right]$$

where *υ_e_* is the effective number of chains in an imperfect network. The evaluation of ΔF_el_ derivative is made by using

(A6) $$\frac{\partial \Delta F_{\mathrm{el}}}{\partial n_{1}}\;=\;\frac{\partial \Delta F_{\mathrm{el}}}{\partial \alpha s}\;\times \;\frac{\partial \alpha s}{\partial n_{1}}$$

The solvent contribution to the total volume of the system was computed by incorporation the molar volume of the solvent χ_1_

(A7) $$\alpha^{3}\;=\;\frac{V}{V_{0}}\;=\;\frac{1}{\upsilon_{2}}\;=\;\frac{V_{0}+\left[\frac{n_{1}\chi_{1}}{N}\right]}{V_{0}}$$

where *V_0_* is the polymer volume and *V*_1_ is the solvent molar volume.

(A8) ∂ΔFel∂n1 = kTυe V1V0 [(υ2) ^1/3 − υ22]

In the equilibrium state the elastic reaction of the network structure, a pressure acting on the swollen gel is sufficient to increase the chemical potential of the solvent in the solution. This action equals the excess solvent surrounding the swollen gel until the equilibrium is reached. The chemical potential of the solvent in the gel is given by:

µ_1_ − µ_1_^0^

(A9) $$\mu_{1}-\mu_{1}^{0}\;=\;N\times \left[\frac{\partial \Delta F_{M}\;}{\partial n_{1}\;}\right]T,\;P\;+\;N\left[\frac{\partial \Delta F_{\mathrm{el}}\;}{\partial \alpha_{s}\;}\right]T,\;P\left[\frac{\partial \alpha s\;}{\partial n_{1}\;}\right]T,\;P$$

where µ_1_ is the chemical potential of the solvent in the gel, $\mu_{1}^{0}$ is the chemical potential in the pure liquid, N is the number of Avogadro.

The derivatives in Equation (A9) were evaluated by using Equations (A3) and (A8).

(A10) μ1−μ10 = NkT×{[ln(1−υ2)+υ2+χ1(υ2)2]+NkTυe×V1V0[(υ2)^1/3−υ22]}

(A10’) μ1−μ10 = NkT×{[ln(1−υ2)+υ2+χ1(υ2)2]+υe×V1V0[(υ2)^1/3−υ22]} 

The first three terms of Equation (A10’) such as *ln* (1−*υ*_2_), *υ*_2_ and *χ*_1_ *υ_2_^2^* express ∂ΔF_M/_∂n_1_ as well as correspond to the lowering of the chemical potential due to mixing of polymer and solvent. The last term [*V_1_ (υ_e/_V_0_*) (*υ_2_^1/3^* − *υ*_2_/2)] describes the increase of the chemical potential from the elastic reaction of the network.

To express *υ_e_* in moles N and k have been replaced by R according to the equation:

(A10’’) R=N×k

(A10’’’) $$\frac{\upsilon_{e}}{V_{0}}=\frac{1}{\tilde{v}M_{c}}$$

(A10’’’’) μ1−μ10=RT×{[ln(1−υ2)+υ2+χ1(υ2)2]+V1v~Mc[(υ2)^1/3−υ22]}

where $\tilde{v}$ is the specific volume of polymer and M_c_ is the molecular weight between the two cross-links. The thermodynamic equilibrium is reached when the chemical potential of the solvent in the polymer equals the pure solvent so that the left side of Equation (A10’’’’) will be equal to zero. Therefore, the Equation (A10’’’’) can be rearranged as follows [54,89].

(A11) −[ln(1−υ2)+υ2+χ1(υ2)2]=V1v~Mc[(υ2)^1/3−υ22]

(A12) Mc=V1v~[(υ2)^1/3−υ22]−[ln(1−υ2) +υ2 + χ1(υ2)2] 

## References

[1] Salimi-Kenari H., Mollaie F., Dashtimoghadam E., Imani M., Nyström B. (2018). Effects of chain length of the cross-linking agent on rheological and swelling characteristics of dextran hydrogels. Carbohydr. Polym..

[2] Hennink W.E., Nostrum C.F. (2012). Van Novel crosslinking methods to design hydrogels. Adv. Drug Deliv. Rev..

[3] Caldera F., Tannous M., Cavalli R., Zanetti M., Trotta F. (2017). Evolution of Cyclodextrin Nanosponges. Int. J. Pharm..

[4] Oliveira V.A., Veloso T.C., Leao V.A. (2013). Hydrogels of cellulose acetate crosslinked with pyromellitic dianhydride-Part I: Synthesis and swelling kinetics. Artigo.

[5] Larrañeta E., Stewart S., Ervine M., Al-Kasasbeh R., Donnelly F.R. (2018). Hydrogels for Hydrophobic Drug Delivery. Classification, Synthesis and Applications. J. Funct. Biomater..

[6] Zhang D., Lv P., Zhou C., Zhao Y., Liao X., Yang B. (2019). Cyclodextrin-based delivery systems for cancer treatment. Mater. Sci. Eng. C.

[7] Swaminathan S., Vavia P.R., Trotta F., Cavalli R. (2013). Nanosponges encapsulating dexamethasone for ocular delivery: Formulation design, physicochemical characterization, safety and corneal permeability assessment. J. Biomed. Nanotechnol..

[8] Mognetti B., Barberis A., Marino S., Berta G., De Francia S., Trotta F., Cavalli R. (2012). In Vitro enhancement of anticancer activity of paclitaxel by a Cremophor free cyclodextrin-based nanosponge formulation. J. Incl. Phenom. Macrocycl. Chem..

[9] Ferro M., Castiglione F., Punta C., Melone L., Panzeri W., Rossi B., Trotta F., Mele A. (2014). Anomalous diffusion of ibuprofen in cyclodextrin nanosponge hydrogels: An HRMAS NMR study. Beilstein J. Org. Chem..

[10] Trotta F., Cavalli R. (2009). Characterization and Applications of New Hyper-Cross-Linked Cyclodextrins. Compos. Interfaces.

[11] Lucia Appleton S., Tannous M., Argenziano M., Muntoni E., Carolina Rosa A., Rossi D., Caldera F., Scomparin A., Trotta F., Cavalli R. (2020). Nanosponges as protein delivery systems: Insulin, a case study. Int. J. Pharm..

[12] Loftsson T., Hreinsdóttir D., Másson M. (2005). Evaluation of cyclodextrin solubilization of drugs. Int. J. Pharm..

[13] Loftsson T., Brewster M.E. (2010). Pharmaceutical applications of cyclodextrins: Basic science and product development. J. Pharm. Pharmacol..

[14] Brewster M.E., Loftsson T. (2007). Cyclodextrins as pharmaceutical solubilizers. Adv. Drug Deliv. Rev..

[15] Saokham P., Muankaew C., Jansook P., Loftsson T. (2018). Solubility of cyclodextrins and drug/cyclodextrin complexes. Molecules.

[16] Del Valle E.M.M. (2004). Cyclodextrins and their uses: A review. Process. Biochem..

[17] Hedges A., BeMiller J., Whistler R. (2009). Cyclodextrins: Properties and Applications. Starch—Chemistry and Technology.

[18] Stella V.J., Rajewski R.A. (1997). Cyclodextrins: Their Future in Drug Formulation and Delivery. Pharm. Res..

[19] Rousseau J., Menuel S., Rousseau C., Hapiot F., Monflier E. (2016). Cyclodextrins as Porous Material for Catalysis.

[20] Hedges A.R. (1998). Industrial applications of cyclodextrins. Chem. Rev..

[21] Trotta F., Dianzani C., Caldera F., Mognetti B., Cavalli R. (2014). The application of nanosponges to cancer drug delivery. Expert Opin. Drug Deliv..

[22] Trotta F. (2011). Cyclodextrin Nanosponges and their Applications. Cyclodextrins in Pharmaceutics, Cosmetics, and Biomedicine.

[23] Szejtli J. (2004). Past, present, and future of cyclodextrin research. Pure Appl. Chem..

[24] Reddy N., Yang Y. (2010). Citric acid cross-linking of starch films. Food Chem..

[25] Rao M., Bajaj A., Khole I., Munjapara G., Trotta F. (2013). In Vitro and in vivo evaluation of β-cyclodextrin-based nanosponges of telmisartan. J. Incl. Phenom. Macrocycl. Chem..

[26] Afinjuomo F., Barclay T.G., Song Y., Parikh A., Petrovsky N., Garg S. (2019). Synthesis and characterization of a novel inulin hydrogel crosslinked with pyromellitic dianhydride. React. Funct. Polym..

[27] Rossi B., Caponi S., Castiglione F., Corezzi S., Fontana A., Giarola M., Mariotto G., Mele A., Petrillo C., Trotta F. (2012). Networking Properties of Cyclodextrin-Based Cross-Linked Polymers Probed by Inelastic Light-Scattering Experiments. J. Phys. Chem. B.

[28] Pawar S., Shende P., Trotta F. (2019). Diversity of β -cyclodextrin-based nanosponges for transformation of actives. Int. J. Pharm..

[29] Shende P., Kulkarni Y.A., Gaud R.S., Deshmukh K., Cavalli R., Trotta F., Caldera F. (2015). Acute and repeated dose toxicity studies of different β-cyclodextrin-based nanosponge formulations. J. Pharm. Sci..

[30] Pushpalatha R., Selvamuthukumar S., Kilimozhi D. (2019). Cyclodextrin nanosponge based hydrogel for the transdermal co-delivery of curcumin and resveratrol: Development, optimization, in vitro and ex vivo evaluation. J. Drug Deliv. Sci. Technol..

[31] Shende P.K., Trotta F., Gaud R.S., Deshmukh K., Cavalli R., Biasizzo M. (2012). Influence of different techniques on formulation and comparative characterization of inclusion complexes of ASA with β-cyclodextrin and inclusion complexes of ASA with PMDA cross-linked β-cyclodextrin nanosponges. J. Incl. Phenom. Macrocycl. Chem..

[32] Bastiancich C., Scutera S., Alotto D., Cambieri I., Fumagalli M., Casarin S., Rossi S., Trotta F., Stella M., Cavalli R. (2015). Cyclodextrin-Based Nanosponges as a Nanotechnology Strategy for Imiquimod Delivery in Pathological Scarring Prevention and Treatment. J. Nanopharm. Drug Deliv..

[33] Loftsson T., Brewster E.M. (1996). Pharmaceutical Applications of Cyclodextrins. 1. Drug Solubilization and Stabilization. J. Pharm. Sci..

[34] Bibby D.C., Davies N.M., Tucker I.G. (2000). Mechanisms by which cyclodextrins modify drug release from polymeric drug delivery systems. Int. J. Pharm..

[35] Hayiyana Z., Choonara Y., Makgotloe A., Toit L., Kumar P., Pillay V. (2017). Ester-Based Hydrophilic Cyclodextrin Nanosponges for Topical Ocular Drug Delivery. Curr. Pharm. Des..

[36] Crupi V., Fontana A., Giarola M., Majolino D., Mariotto G., Mele A., Melone L., Punta C., Rossi B., Venuti V. (2013). Connection between the vibrational dynamics and the cross-linking properties in cyclodextrins-based polymers. J. Raman Spectrosc..

[37] Cesar Hernandez-Ortiz J., Vivaldo-lima E., Saldivar-Guerra E., Vivaldo-Lima E. (2013). Crosslinking. Handbook of Polymer Synthesis, Characterization and Processing.

[38] Akalp U., Chu S., Skaalure S.C., Bryant S.J., Doostan A., Vernerey F.J. (2015). Determination of the polymer-solvent interaction parameter for PEG hydrogels in water: Application of a self learning algorithm. Polymer.

[39] Krabicová I., Appleton S.L., Tannous M., Hoti G., Caldera F., Pedrazzo A.R., Cecone C., Cavalli R., Trotta F. (2020). History of cyclodextrin nanosponges. Polymers.

[40] Li D., Ma M. (2000). Nanosponges for water purification. Clean Prod. Process..

[41] Cecone C., Hoti G., Krabicova I., Appleton S.L., Caldera F., Bracco P., Zanetti M., Trotta F. (2020). Sustainable synthesis of cyclodextrin-based polymers exploiting natural deep eutectic solvents. Green Chem..

[42] Pedrazzo A.R., Caldera F., Zanetti M., Appleton S.L., Dahkar N.K., Trotta F. (2020). Mechanochemical green synthesis of hyper-crosslinked cyclodextrin polymers. Beilstein J. Org. Chem..

[43] Mele A., Castiglione F., Malpezzi L., Ganazzoli F., Raffaini G., Trotta F., Rossi B., Fontana A., Giunchi G. (2011). HR MAS NMR, powder XRD and Raman spectroscopy study of inclusion phenomena in bCD nanosponges. J. Incl. Phenom. Macrocycl. Chem..

[44] Castiglione F., Crupi V., Majolino D., Mele A., Rossi B., Trotta F., Venuti V. (2012). Effect of Cross-Linking Properties on the Vibrational Dynamics of Cyclodextrins-Based Polymers: An Experimental–Numerical Study. J. Phys. Chem. B.

[45] Rossi B., Paciaroni A., Venuti V., Fadda G.C., Melone L., Punta C., Crupi V., Majolino D., Mele A. (2017). SANS investigation of water adsorption in tunable cyclodextrin-based polymeric hydrogels. Phys. Chem. Chem. Phys..

[46] Zanetti M., Anceschi A., Magnacca G., Spezzati G., Caldera F., Rosi G.P., Trotta F. (2016). Micro porous carbon spheres from cyclodextrin nanosponges. Microporous Mesoporous Mater..

[47] Trotta F., Tumiatti W., Vallero R. (2004). Nanospugne a Base di Ciclodestrine Funzionalizzate con Gruppi Carbossilici: Sintesi e Utilizzo Nella Decontaminazione da Metalli Pesanti e da Composti Organici, Separazioni Cromatografiche e Veicolazione di Farmaci. Italian Patent.

[48] Wong R.S.H., Ashton M., Dodou K. (2015). Effect of crosslinking agent concentration on the properties of unmedicated hydrogels. Pharmaceutics.

[49] Schreiber H.P., Holden H.W., Barna G. (1970). Rapid determination of crosslink densities and interaction parameters from swelling rate data. J. Polym. Sci., Part. C.

[50] Witono J.R., Noordergraaf I., Heeres H.J., Janssen L.P.B.M., Heeres H. (2014). Water absorption, retention and the swelling characteristics of cassava starch grafted with polyacrylic acid. Carbohydr. Polym..

[51] Kuang J., Yuk K.Y., Huh K.M. (2011). Polysaccharide-based superporous hydrogels with fast swelling and superabsorbent properties. Carbohydr. Polym..

[52] Neuburger N.A., Eichinger B.E. (1988). Critical Experimental Test of the Flory-Rehner Theory of Swelling. Macromolecules.

[53] Peppas N.A., Huang Y., Torres-Lugo M., Ward J.H., Zhang J. (2000). Physicochemical Foundations and Structural Design of Hydrogels in Medicine and Biology. Annu. Rev. Biomed. Eng..

[54] Flory P.J. (1953). Principles of Polymer Chemistry.

[55] Chassé W., Lang M., Sommer J.-U., Saalwächter K. (2011). Cross-Link Density Estimation of PDMS Networks with Precise Consideration of Networks Defects. Macromolecules.

[56] Xia Z., Patchan M., Maranchi J., Elisseeff J., Trexler M. (2013). Determination of Crosslinking Density of Hydrogels Prepared from Microcrystalline Cellulose. J. Appl. Polym. Sci..

[57] Riedo C., Caldera F., Poli T., Chiantore O. (2015). Poly(vinylalcohol)-borate hydrogels with improved features for the cleaning of cultural heritage surfaces. Herit. Sci..

[58] Bossard F., Aubry T., Gotzamanis G., Tsitsilianis C. (2006). pH-Tunable rheological properties of a telechelic cationic polyelectrolyte reversible hydrogel. R. Soc. Chem..

[59] Durairaj R., Wai Man L., Ekere N.N., Mallik S. (2010). The effect of wall-slip formation on the rheological behaviour of lead-free solder pastes. Mater. Design.

[60] Shakeel A., Kirichek A., Chassagne C. (2020). Effect of pre-shearing on the steady and dynamic rheological properties of mud sediments. Mar. Pet. Geol..

[61] Kulicke W.-M., Aggour Y.A., Nottelmann H., Elsabee M.Z. (1989). Swelling and Rheological Studies of Some Starch Hydrogels. Starch-Stärke.

[62] Yang S.-Y., Yuan L.-L. (2018). Advanced Polyimide Films. Advanced Polyimide Materials, Synthesis, Characterization and Application.

[63] Tokita M. (2014). Structure and Frictional Properties of Colloid Gel. Polymers.

[64] Doi Y., Tokita M. (2005). Real Space Structure of Opaque Gel. Am. Chem. Soc..

[65] Kowalski G., Kijowska K., Witczak M., Kuterasiński Ł., Łukasiewicz M. (2019). Synthesis and Effect of Structure on Swelling Properties of Hydrogels Based on High Methylated Pectin and Acrylic Polymers. Polymers.

[66] Sarin V.K., Kent S.B.H., Merrifield R.B. (1980). Properties of Swollen Polymer Networks. Solvation and Swelling of Peptide-Containing Resins in Solid-Phase Peptide Synthesis. J. Am. Chem. Soc..

[67] Flory P.J., Rehner J. (1943). Statistical Mechanics of Cross-Linked Polymer Networks II. Swelling. J. Chem. Phys..

[68] Pó R. (1994). Water-Absorbent Polymers: A Patent Survey. J. Macromol. Sci..

[69] Van Vlierberghe S., Graulus G., Samal S.K., Van Nieuwenhove I., Dubruel P. (2014). Porous Hydrogel Biomedical foam Scaffolds for Tissue Repair.

[70] Flory P.J., Rehner J. (1943). Statistical Theory of Chain Configuration and Physical Properties of High Polymers. Ann. New York Acad. Sci..

[71] Valentín J.L., Carretero-González J., Mora-Barrantes I., Chassé W., Saalwächter K. (2008). Uncertainties in the determination of cross-link density by equilibrium swelling experiments in natural rubber. Macromolecules.

[72] Quesada-Pérez M., Maroto-Centeno J.A., Forcada J., Hidalgo-Alvarez R. (2011). Gel swelling theories: The classical formalism and recent approaches. Soft Matter.

[73] Fennell E., Kamphus J., Huyghe J.M. (2020). The Importance of the Mixing Energy in Ionized Superabsorbent Polymer Swelling Models. Polymers.

[74] Urich M., Denton A.R. (2016). Swelling, structure, and phase stability of compressible microgels. R. Soc. Chem..

[75] Fennell E., Huyghe J.M. (2019). Chemically Responsive Hydrogel Deformation Mechanics: A Review. Molecules.

[76] Camponeschi F., Atrei A., Rocchigiani G., Mencuccini L., Uva M., Barbucci R. (2015). New Formulations of Polysaccharide-Based Hydrogels for Drug Release and Tissue Engineering. Gels.

[77] Pasqui D., De Cagna M., Barbucci R. (2012). Polysaccharide-Based Hydrogels: The Key Role of Water in Affecting Mechanical Properties. Polymers.

[78] Hajighasem A., Kabiri K. (2013). Cationic highly alcohol-swellable gels: Synthesis and characterization. J. Polym. Res..

[79] Grillet A.M., Wyatt N.B., Gloe L.M., De Vicente J. (2012). Polymer Gel Rheology and Adhesion. Rheology.

[80] Carvalho J., Moreira S., Maia J., Gama F.M. (2010). Characterization of dextrin-based hydrogels: Rheology, biocompatibility, and degradation. J. Biomed. Mater. Res. Part A.

[81] Romania F., Corrieri R., Bragab V., Ciardelli F. (2002). Monitoring the chemical crosslinking of propylene polymers through rheology. Polymer.

[82] Schurz J. (1996). Rheology of synovial fluids and substitute polymers. J. Macromol. Sci. Pure Appl. Chem..

[83] Cardinaels R., Reddy N.K., Clasen C. (2019). Quantifying the errors due to overfilling for Newtonian fluids in rotational rheometry. Rheol. Acta.

[84] Tanaka F., Ishida M. (1996). Elastically Effective Chains in Transient Gels with Multiple Junctions. Macromolecules.

[85] Kulicke W.M., Nottelmann H. (1989). Structure and Swelling of Some Synthetic, Semisynthetic and Biopolymer Hydrogels. Polym. Aqueous Media; Glas. J. Adv. Chem. Am. Chem. Soc..

[86] Sombatsompop N. (1998). Practical Use of the Mooney-Rilvin Equation for Determination of Degree of Cross-linking of Swollen NR Vulcanisates. J. Sci. Soc. Thail..

[87] Wood L.A. (1979). Molecular Interpretations of Modulus and Swelling Relations in Natural Rubber Cross-Linked by Dicumyl Peroxide. J. Res. Natl. Bur. Stand..

[88] Lopez C.G., Richtering W. (2017). Does Flory-Rehner theory quantitatively describe the swelling of thermoresposnive microgels?. R. Soc. Chem..

[89] Bray J.C., Merrill E.W. (1973). Poly (vinyl Alcohol) Hydrogels. Formation by Electron Beam Irradiation. J. Appl. Polym. Sci..

