# Supplementary material for: Effect of the Cross-Linking Density on the Swelling and Rheological Behavior of Ester-Bridged β-Cyclodextrin Nanosponges
Source: Materials (Basel). 2021 Jan 20;14(3):478. doi: 10.3390/ma14030478 (PMC7864023; doi:10.3390/ma14030478)
Supplement: Supplementary file 1 [file materials-14-00478-s001.pdf]

materials

MDPI

Supplementary Materials

Effect of the Cross-Linking Density on the Swelling and Rheological Behavior of Ester-Bridged β-Cyclodextrin Nanosponges

Gjylije Hoti*, Fabrizio Caldera, Claudio Cecone, Alberto Rubin Pedrazzo, Anastasia Anceschi, Silvia Lucia Appleton, Yousef Khazaei Monfared and Francesco Trotta

S1. Polymer Network

A polymer network is defined as a cross-linked macromolecule which has all units connected via chemical or physical cross-linking [1].

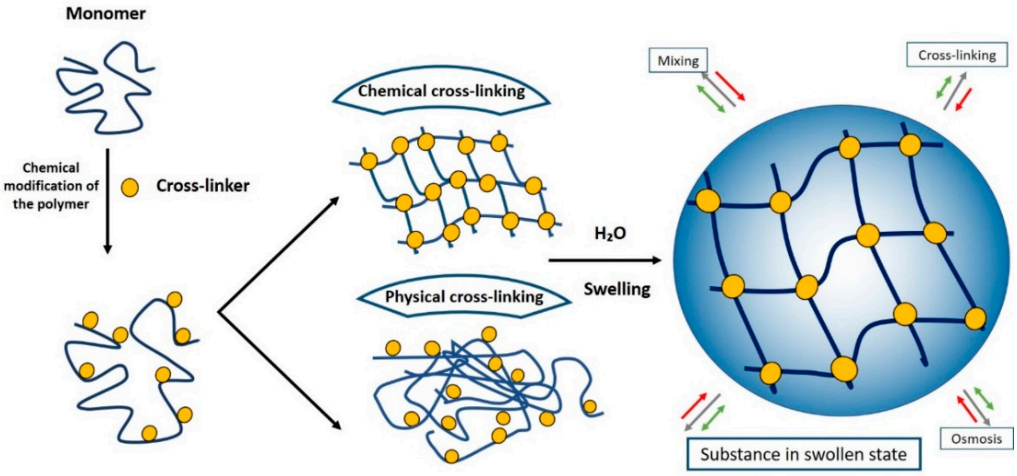

Figure S1. Chemical modification of the polymer.

S2. Experimental Setup

During the experiment, β-CD:PMDA molar ratio is a parameter that was altered in order to investigate the influence of PMDA as a cross-linker in the NS formation, as it is detailed in Table S1.

Citation: Hoti, G.; Caldera, F.; Cecone, C.; Rubin Pedrazzo, A.; Anceschi, A.; Appleton, S.L.; Monfared, Y.K.; Trotta, F. Effect of the Cross-Linking Density on the Swelling and Rheological Behavior of Ester-Bridged β-Cyclodextrin Nanosponges. *Materials* **2020**, *14*, 478. https://doi.org/10.3390/ma14030478

Received: 21 December 2020
Accepted: 8 January 2021
Published: 20 January 2021

Publisher's Note: MDPI stays neutral with regard to jurisdictional claims in published maps and institutional affiliations.

Copyright: © 2021 by the authors. Submitted for possible open access publication under the terms and conditions of the Creative Commons Attribution (CC BY) license (http://creativecommons.org/licenses/by/4.0/).

Table S1. The varying amounts of PMDA as a cross-linker in the synthesis of β-CD NSs.

Molar Ratio (β-CD:PMDA)	β-CD (g)	PMDA (g)	Molar Ratio (Glucose Units: PMDA)
1:2		1.87	1:0.285
1:3		2.81	1:0.428
1:4		3.75	1:0.571
1:5		4.69	1:0.714
1:6	4.886	5.63	1:0.857
1:7		6.57	1:1.000
1:8		7.51	1:1.142
1:9		8.45	1:1.285
1:10		9.38	1:1.428

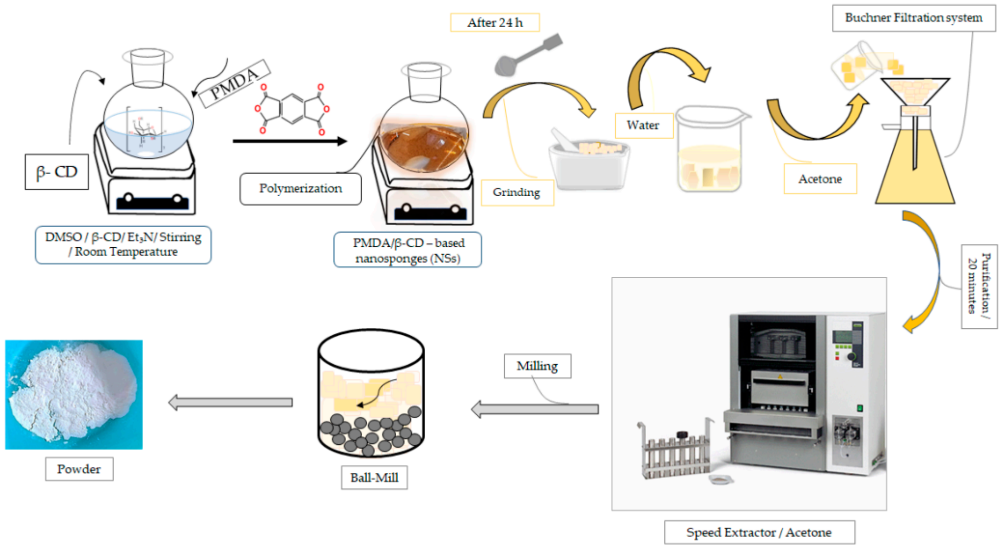

Figure S2. Scheme of the synthesis of β-CD:PMDA NSs.

Figure S2 illustrates the experimental setup of β-CD:PMDA NSs synthesis.

S3. Swelling Studies

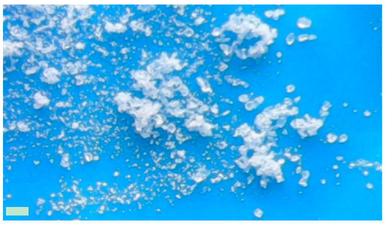

1:4 (**A**)

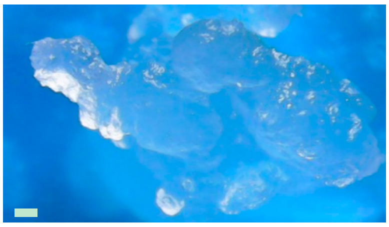

1:4 (**B**)

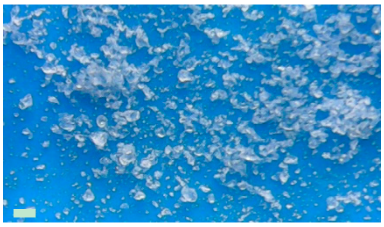

1:5 (**A**)

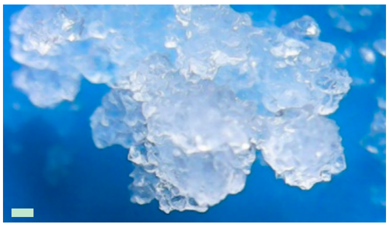

1:5 (**B**)

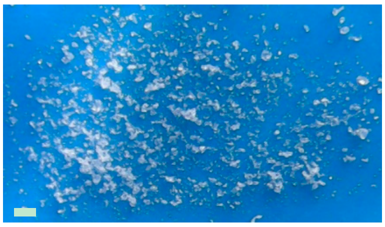

1:7 (**A**)

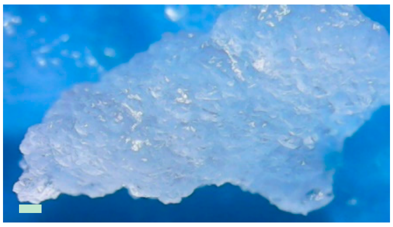

1:7 (**B**)

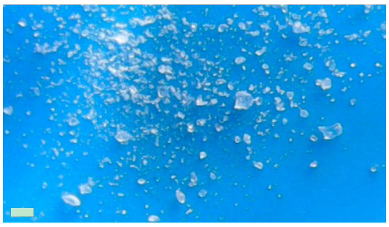

1:8 (**A**)

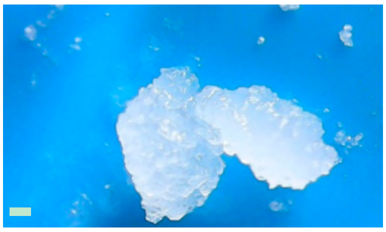

1:8 (**B**)

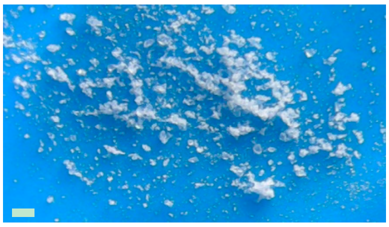

1:9 (**A**)

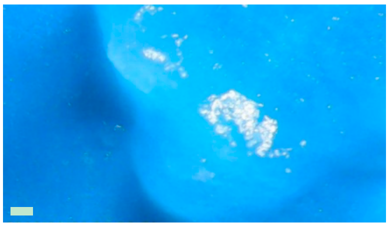

1:9 (**B**)

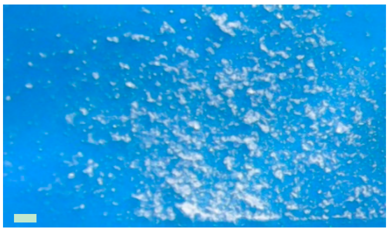

1:10 (**A**)

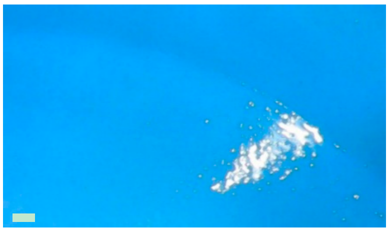

1:10 (**B**)

Figure S3. Images of β-CD:PMDA NS molar ratio in a dry state (**A**) and in a swollen state (**B**).

S4. Density of Polymer

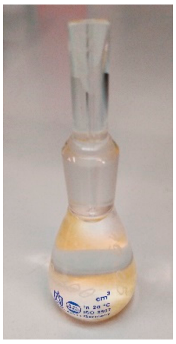

Figure S4. Pycnometer.

The density of polymer was precisely determined by a calibrated pycnometer equipped with a close-fitting ground glass stopper with a capillary hole through it. After closing a top-filled pycnometer, this fine hole releases a spare liquid. It allows to measure the volume of working liquids with high accuracy [2]. As the β-CD:PMDA NSs can absorb large amounts of water, acetone was used as a working liquid. Firstly, the empty pycnometer and the pycnometer filled with acetone were weighed. Then, by subtracting the latter from the former, the mass of acetone was recorded. The volume of acetone, that filled the pycnometer and the capillary tube, was already known, therefore, the density of acetone was calculated as follows:

$$\rho(acetone) = \frac{m(acetone)}{V(acetone)} \quad (1S)$$

Secondly, the weight of the pycnometer, with the polymer added, was measured. By subtracting the empty pycnometer weight from this value, the mass of the powder was recorded. Furthermore, the weight of the pycnometer, containing polymer and acetone, was measured to evaluate the mass of acetone. Then, the volume of acetone was calculated as follows:

$$V\ (acetone) = \frac{m(acetone)}{\rho(acetone)} \quad (2S)$$

By subtracting this value from the volume of the pycnometer, the volume of powder was obtained:

$$V\ (powder) = V\ (pycnometer) - V\ (acetone) \quad (3S)$$

Lastly, the density of the polymer was calculated:

$$\rho(polymer) = \frac{m(polymer)}{V(polymer)} \quad (4S)$$

S5. Density of Gel

The density of gel was determined following the preceding procedure. Separately, the working liquid was water.

Table S2. Calculated densities of both, the gel and powder of β-CD: PMDA NSs having the various amount of PMDA. Mean Values ± SD.

Molar Ratio (β-CD:PMDA)	Gel Density (g/cm^3)	Polymer Density (g/cm^3)
1:2	1.003	1.44 ± 0.1064
1:3	1.02 ± 0.0043	1.47 ± 0.035
1:4	1.01 ± 0.0090	1.48 ± 0.024
1:5	1.23 ± 0.0247	1.53 ± 0.045
1:6	1.17 ± 0.0088	1.34 ± 0.2745
1:7	1.21 ± 0.0144	1.42 ± 0.1537
1:8	1.21 ± 0.0280	1.29 ± 0.0858
1:9	1.14 ± 0.0066	1.54 ± 0.1060
1:10	1.12 ± 0.0206	1.48 ± 0.0742

S6. Sample Loading

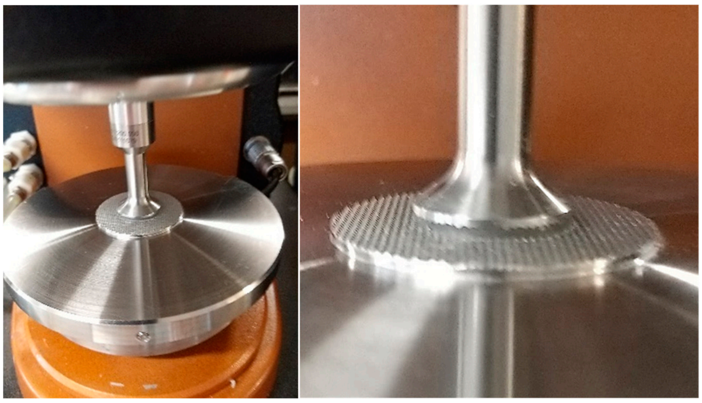

Figure S5. Sample loading in a rheometer, 1 mm gap size, removal of the extra sample outside the geometry and of the solvent trap.

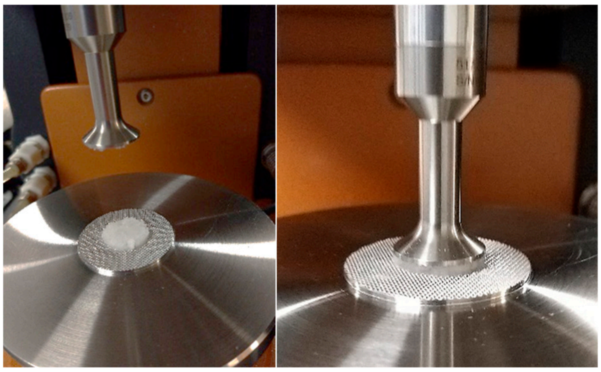

Figure S6. Sample loading in a rheometer, 2 mm gap size, removal of the extra sample outside the geometry and of the solvent trap.

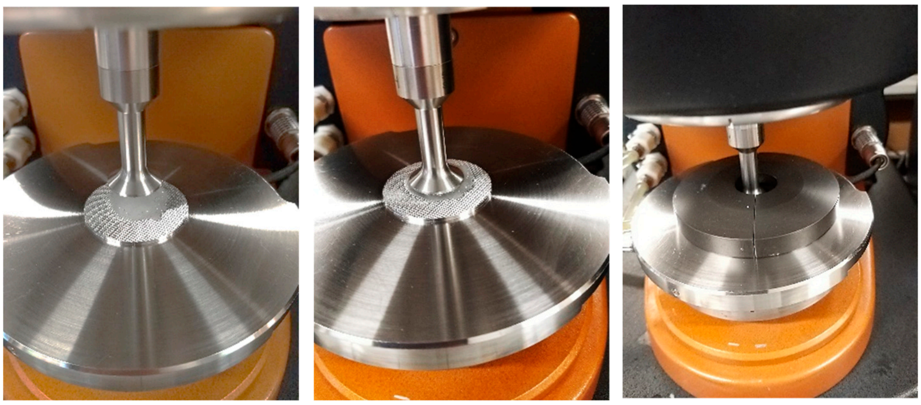

Figure S7. Sample loading in a rheometer, 1 mm gap size. Non-removal of the extra sample outside the geometry and of the solvent trap.

S7. Water Absorption Capacity (WAC)

As described in the article, the swelling or water absorption capacity (WAC) of the β-CD:PMDA NSs is inversely proportional to the content of PMDA. The WAC experimental values are between 158-1526 g H_2O/g dry sample.

Table S3. (**a**) WAC experimental values of β-CD:PMDA NSs; (**b**), (**c**) Water absorption capacity (WAC) as the function of the swelling time to monomer ratio of β-CD:PMDA NSs.

(**a**)

Molar Ratio (β-CD:PMDA)	WAC (%)
1:2	1526 ± 76.18
1:3	691 ± 80.03
1:4	241 ± 2.24
1:5	174 ± 4.38
1:6	165 ± 1.44
1:7	158 ± 1.38
1:8	159 ± 6.46
1:9	162 ± 5.41
1:10	218 ± 16.34

(**b**)

β-CD:PMDA Molar Ratio	WAC (%) 0.5 h	WAC (%) 2 h	WAC (%) 4 h	WAC (%) 6 h
1:2	1527 ± 29.666	2084 ± 120.083	2110 ± 52.706	1988 ± 78.450
1:3	468 ± 11.099	625 ± 4.915	651 ± 35.610	712 ± 25.758
1:4	245 ± 17.758	266 ± 5.505	272 ± 7.976	264 ± 4.112
1:5	160 ± 1.426	181 ± 11.091	171 ± 6.402	189 ± 12.232
1:6	156 ± 2.683	166 ± 8.513	155 ± 8.531	159 ± 8.827
1:7	147 ± 1.39	158 ± 9.729	152 ± 1.577	156 ± 15.861
1:8	163 ± 4.195	169 ± 3.88	171 ± 1.830	181 ± 0.758
1:9	174 ± 0.46	161 ± 25.42	176 ± 7.098	191 ± 3.74
1:10	204 ± 7.305	240 ± 1.75	258 ± 0.875	261 ± 14.119

(**c**)

β-CD:PMDA Molar Ratios	WAC (%) 12 h	WAC (%) 24 h	WAC (%) 48 h	WAC (%) 72 h
1:2	1808 ± 187.1	0	0	0
1:3	775 ± 26.466	755 ± 7.7	924 ± 37.638	988 ± 9.428
1:4	275 ± 3.603	275 ± 3.577	316 ± 13.867	372 ± 5.72
1:5	168 ± 5.8	163 ± 4.68	188 ± 1.573	192 ± 7.391
1:6	146 ± 0.4	142 ± 2.205	150 ± 4.121	159 ± 4.147
1:7	140 ± 11.21	138 ± 5.401	155 ± 6.058	165 ± 6.637
1:8	166 ± 2.003	152 ± 3.596	188 ± 1.635	217 ± 21.699
1:9	206 ± 3.24	200 ± 3.4824	192 ± 0.645	241 ± 8.189
1:10	298 ± 2.83	307 ± 4.780	314 ± 3.009	390 ± 6.476

S8. Flory-Rehner Theory

Table S4. Calculated physicochemical terms (M_c, v, $v_{2,m}$) of β-CD:PMDA NSs having the various amount of PMDA. Mean Values ± SD.

Molar Ratios (β-CD:PMDA)	Polymer Volume Fraction in the Swollen Mass (v_{2m})	Cross-linking Density ((v), mol/cm³)	Molecular Weight Between Cross-links ((M_c), g/mol)
1:2	0.05042 ± 0.00312	3.14 E-5 ± 5.95E-6	46975.15 ± 8145.33
1:3	0.09214 ± 0.00463	9.63 E-5 ± 3.1 E-5	13218.14 ± 1612.74
1:4	0.21423 ± 0.01062	9.47 E-4 ± 9.6 E-5	1562.71 ± 223.61
1:5	0.23898 ± 0.01452	0.00137 ± 2.35 E-4	1155.73 ± 197.29
1:6	0.26294 ± 0.00531	0.00171 ± 9.93 E-5	869.14 ± 51.31
1:7	0.26336 ± 0.00861	0.00168 ± 2.16 E-4	867.14 ± 83.29
1:8	0.26923 ± 0.0131	0.00178 ± 2.47 E-4	816.71 ± 110.40
1:9	0.26278 ± 0.00736	0.00167 ± 2.17 E-4	871.92 ± 72.61
1:10	0.17692 ± 0.01055	5.25 E-4 ± 1.75 E-4	2626.39 ± 394.66

S9. Rheological Measurements

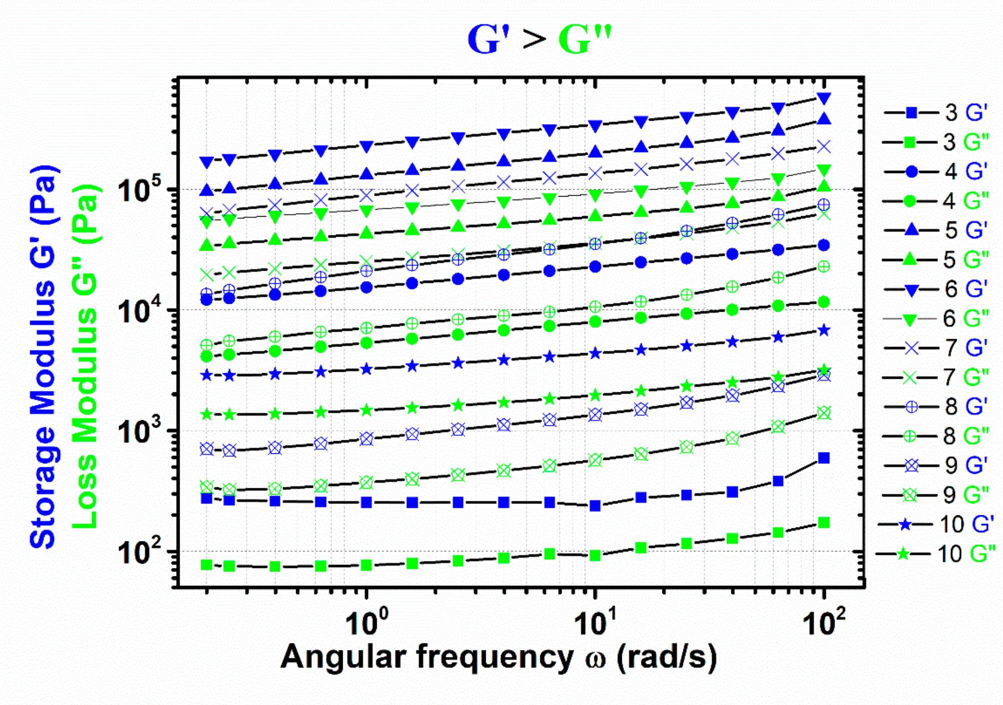

Figure S8. Storage (G′) and loss (G′′) modulus versus angular frequency for β-CD:PMDA molar ratio of 1:3, 1:4, 1:5, 1:6, 1:7, 1:8, 1:9, 1:10. 1 mm gap size without removing the extra sample outside the geometry and with solvent trap.

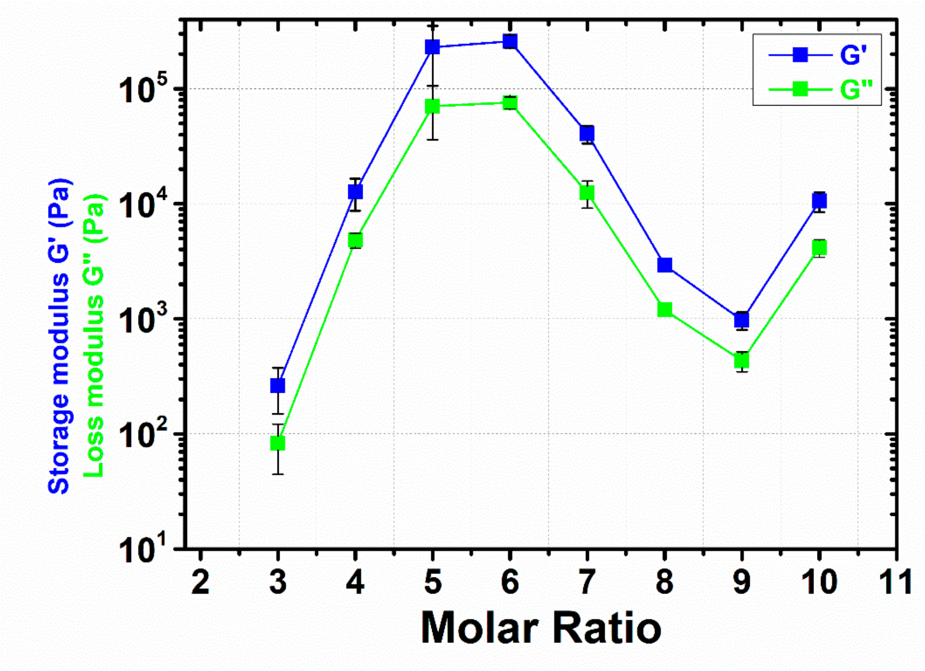

Figure S9. Storage (G′) and loss (G′′) modulus versus molar ratio of β-CD:PMDA (1:3, 1:4, 1:5, 1:6, 1:7, 1:8, 1:9, 1:10) at an angular frequency (ω) of 1 rad/s; 1 mm gap size without removing the extra sample outside the geometry and with solvent trap.

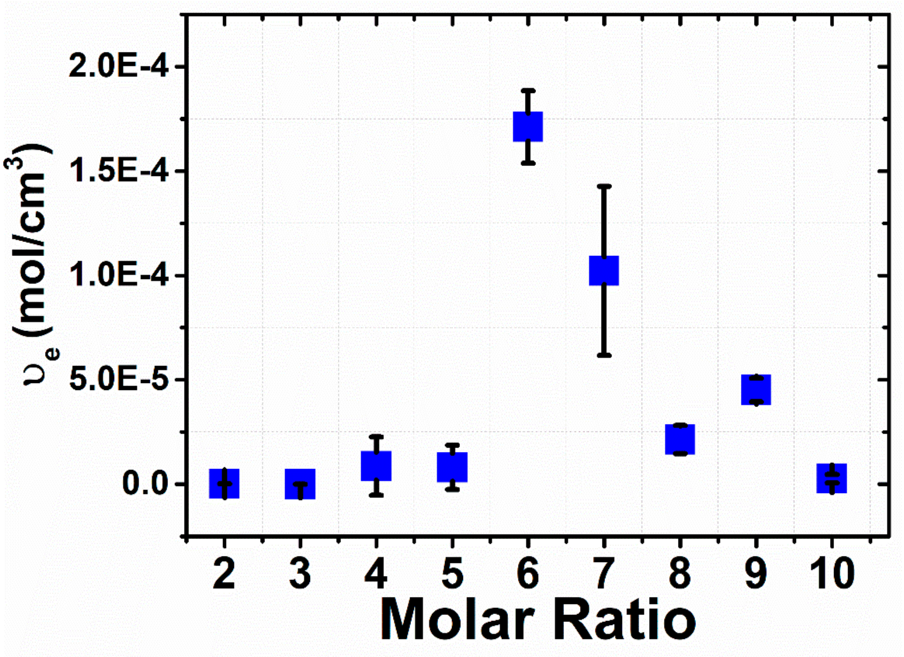

Figure S10. Effective sub-chain density (moles of effective sub-chains per unit volume, υ_e, mol/cm^3) as a function of added cross-linker content. 1 mm gap size without removing the extra sample outside the geometry and with solvent trap.

Table S5. Calculated rheological parameters of β-CD:PMDA NSs having various amount of PMDA. Mean Values ± SD. Gap size (1 mm) without removing the extra sample outside the geometry and with solvent trap.

Molar Ratio (β-CD:PMDA)	G′ (Pa)	G″ (Pa)	υ_e (mol/cm^3),
1:3	262.667 ± 113.249	82.9 ± 38.45	3.64 E-7 ± 1.49 E-7
1:4	12630 ± 3940	4820 ± 707.10	2.17 E-5 ± 4.88 E-6
1:5	229030 ± 122830	70590 ± 34640	4.94 E-5 ± 4.52E-6
1:6	258580 ± 33710	75750 ± 8970	8.91 E-5 ± 8.21 E-6
1:7	40500 ± 7080	12460 ± 3290	1.02 E-4 ± 1.9 E-5
1:8	8120 ± 11270	2810 ± 3730	7.04 E-5 ± 1.03 E-5
1:9	2990 ± 3500	1220 ± 1360	1.84 E-5 ± 9.72 E-6
1:10	10530 ± 2040	4150 ± 723.901	1.62 E-5 ± 3.33 E-6

Table S6. Calculated rheological parameters of β-CD:PMDA NSs having various amount of PMDA. Mean Values ± SD. Gap size (1 mm) removing the extra sample outside the geometry and without solvent trap.

Molar Ratio (β-CD:PMDA)	G′ (Pa)	G″ (Pa)	υ_e (mol/cm^3),
1:2	165.058 ± 46.99	47.971 ± 10.691	2.17 E-7 ± 8.12 E-8
1:3	17.721 ± 14.54	15.799 ± 17.267	5.19 E-8 ± 2.05 E-9
1:4	442.145 ± 100.138	207.517 ± 46.03	8.68 E-6 ± 1.40 E-5
1:5	1515.905 ± 115.958	595.311 ± 146.14	8.119 E-6 ± 1.06 E-5
1:6	83694.7 ± 22319.38	36070.1 ± 11961.74	1.711 E-4 ± 1.74 E-5
1:7	58090.85 ± 23485.77	22877.5 ± 8518.65	1.022 E-4 ± 4.01 E-5
1:8	11734.74 ± 3504.73	5265.195 ± 1649.78	2.139 E-5 ± 6.68 E-6
1:9	22933.33 ± 3558.74	9688.196 ± 4368.38	4.51 E-5 ± 5.59 E-6
1:10	1843.789 ± 1408.57	584.4515 ± 279.85	2.655 E-6 ± 2.09 E-6

Table S7. Calculated rheological parameters of β-CD:PMDA NSs having various amount of PMDA. Mean Values ± SD. Gap size (2 mm) removing the extra sample outside the geometry and without solvent trap.

Molar Ratio (β-CD:PMDA)	G′ (Pa)	G″ (Pa)	υ_e (mol/cm^3),
1:2	73.104 ± 32.01	23.12 ± 9.13	1.042 E-7 ± 4.46 E-8
1:3	819.38	249.83	1.255 E-6
1:4	5122.1 ± 940.28	1188.7 ± 249.25	7.586 E-6 ± 1.44 E-6
1:5	4545.2 ± 3354.39	1442.8 ±1051.56	1.02 E-5 ± 3.17 E-6
1:6	20579.9 ± 12626.6	10050.4 ± 6583.4	3.84 E-5 ± 9.85 E-6
1:7	16120.4 ± 46.03	7326.945 ± 120.41	2.65 E-5 ± 6.41 E-6
1:8	5061.6 ± 1810.0	2382.28 ± 847.09	9.15 E-6 ± 3.36 E-6
1:9	6362.17 ± 2520.5	2294.44 ± 981.94	8.82 E-6 ± 1.52 E-6
1:10	5138.3 ± 1221.9	1199.75 ± 242.34	7.74 E-6 ± 1.80 E-6

Table S8 and Figure S11 present the comparison of the cross-linking density determination based on different rheological procedures. 1 mm b) presents the rheological procedure carried out using gap size (1 mm) removing the extra sample outside the geometry and without solvent trap (Figure S5), 2 mm presents the rheological procedure carried out using gap size (2 mm) removing the extra sample outside the geometry and without solvent trap (Figure S6), whereas 1 mm a) presents the rheological procedure carried out using gap size (1 mm) without removing the extra sample outside the geometry and solvent trap (Figure S7).

Table S8. Calculated physicochemical term (υ_e) of β-CD:PMDA NSs molar ratio for different rheological procedures as previously described.

Molar Ratio (β-CD: PMDA)	1 mm a)	1 mm b)	2 mm
1:2	-	2.17 E-7	1.042 E-7
1:3	3.64 E-7	5.19 E-8	1.255 E-6
1:4	2.17 E-5	8.68 E-6	7.586 E-6
1:5	4.94 E-5	8.11 E-6	1.02 E-5
1:6	8.91 E-5	1.71 E-4	3.84 E-5
1:7	1.02 E-4	1.02 E-4	2.65 E-5
1:8	7.04 E-5	2.13 E-5	9.15 E-6
1:9	1.84 E-5	4.51 E-5	8.82 E-6
1:10	1.62 E-5	2.65 E-6	7.74 E-6

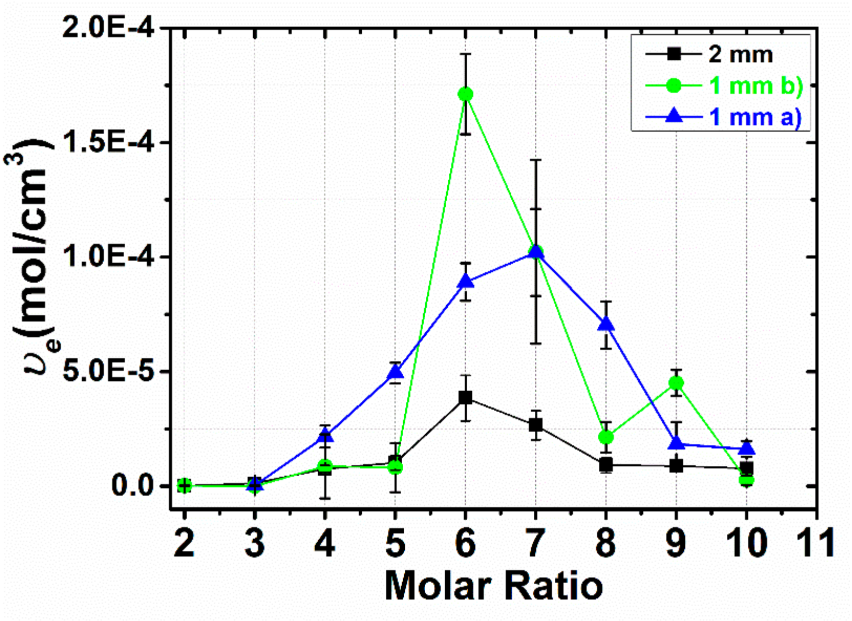

Figure S11. Effective sub-chain density (moles of effective sub-chains per unit volume, υ_e, mol/cm^3) as a function of added cross-linker content, for different rheological procedures as previously described (1 mm a), 1 mm b), 2 mm).

S10. Various Applications of β-CD:PMDA NSs

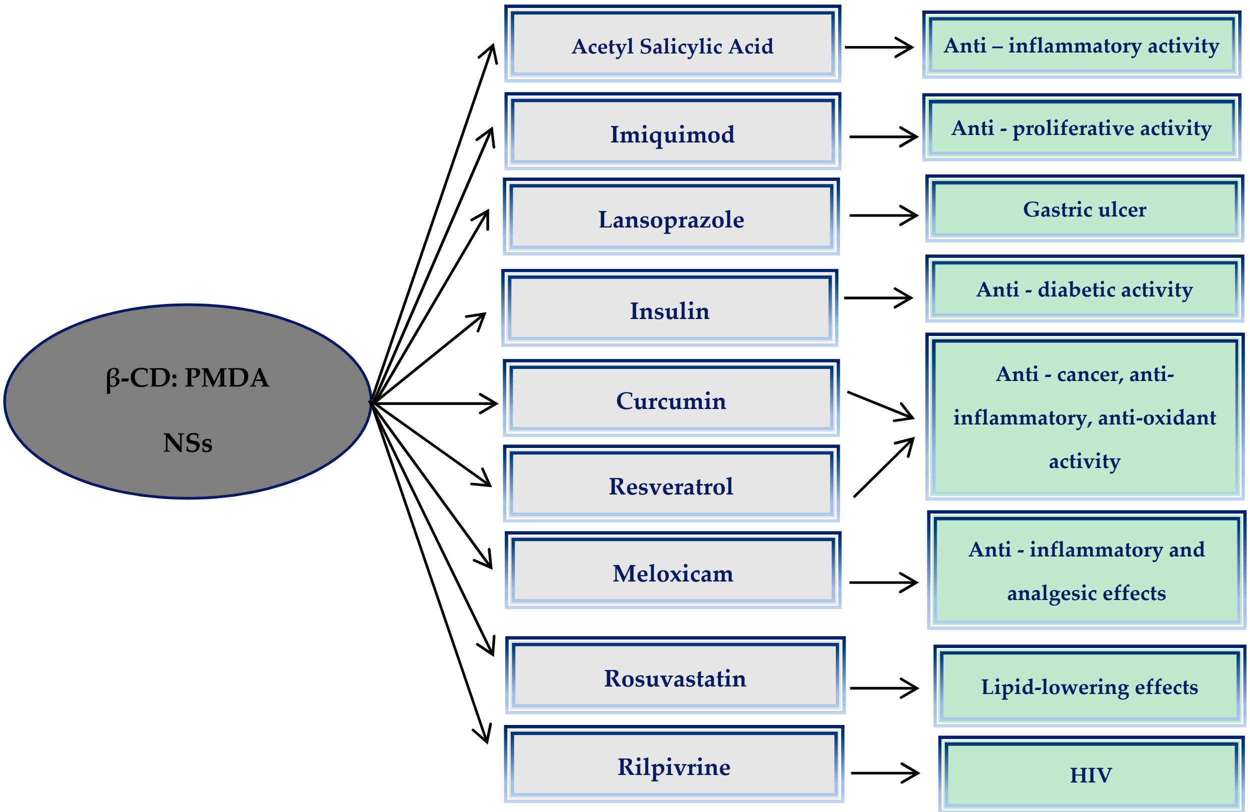

Figure S12. β-CD: PMDA NSs as delivery systems: acetyl salicylic acid [3], imiquimod [4], lansoprazole [5], insulin [6], curcumin [7], resveratrol [8], meloxicam [9], rosuvastatin [10], rilpivrine [11].

References

1. Cesar Hernandez-Ortiz, J.; Vivaldo-lima, E. Crosslinking. In *Handbook of Polymer Synthesis, Characterization and Processing*, 1st ed.; Saldivar-Guerra, E., Vivaldo-Lima, E., Eds.; John Wiley & Sons, Inc., Hoboken, NJ, USA: **2013**; 187–204.
2.. Semnani D. Geometrical Characterization of Electrospun Nanofibers. In *Electrospun Nanofibers*. *Woodhead Publishing Series in Textiles*, Elsevier Ltd, USA, **2017**; 1 – 622.
3. Shende, P.K.; Trotta, F.; Gaud, R.S.; Deshmukh, K.; Cavalli, R.; Biasizzo, M. Influence of different techniques on formulation and comparative characterization of inclusion complexes of ASA with β-cyclodextrin and inclusion complexes of ASA with PMDA cross-linked β-cyclodextrin nanosponges. *J. Incl. Phenom. Macrocycl. Chem.* **2012**, *74*, 447–454.
4. Bastiancich C, Scutera S, Alotto D, et al. Cyclodextrin-Based Nanosponges as a Nanotechnology Strategy for Imiquimod Delivery in Pathological Scarring Prevention and Treatment. *J Nanopharmaceutics Drug Deliv.* **2015**;2(4):311-324.
5. Shende P, Chaphalkar R, Deshmukh K, Gaud RS. Physicochemical Investigation of Engineered Nanosuspensions Containing Model Drug, Lansoprazole. *J Dispers Sci Technol*. **2016**;37(4):504-511.
6. Lucia Appleton S, Tannous M, Argenziano M, et al. Nanosponges as protein delivery systems : Insulin , a case study. *Int J Pharm.* **2020**;590:1-11.
7. Pushpalatha R, Selvamuthukumar S, Kilimozhi D. Cross-linked, cyclodextrin-based nanosponges for curcumin delivery - Physicochemical characterization, drug release , stability and cytotoxicity. *J Drug Deliv Sci Technol.* **2018**;45:45-53.
8. Pushpalatha R, Selvamuthukumar S, Kilimozhi D. Cyclodextrin nanosponge based hydrogel for the transdermal co-delivery of curcumin and resveratrol : Development , optimization , in vitro and ex vivo evaluation. *J Drug Deliv Sci Technol.* **2019**;52:55-64.
9. Shende PK, Gaud RS, Bakal R, Patil D. Effect of inclusion complexation of meloxicam with β-cyclodextrin- and β-cyclodextrin-based nanosponges on solubility, in vitro release and stability studies. *Colloids Surfaces B Biointerfaces*. **2015**;136:105-110.
10. Gabr MM, Mortada SM, Sallam MA. Carboxylate cross-linked cyclodextrin: a nanoporous scaffold for enhancement of rosuvastatin oral bioavailability. *Eur J Pharm Sci*. **2017**:1-47.
11. Rao MRP, Chaudhari J, Trotta F, Caldera F. Investigation of Cyclodextrin-Based Nanosponges for Solubility and Bioavailability Enhancement of Rilpivirine. *AAPS PharmSciTech*. **2018**: 1-12.
